# Multimodal characterization of the responsiveness of eight hepatitis D virus genotype isolates to interferon-alpha treatment

**DOI:** 10.1128/jvi.01280-25

**Published:** 2025-09-18

**Authors:** Yibo Ding, Qiudi Li, Yunlu Sha, Ruilin Si, Chengqian Feng, Mei Liu, Zhanghao Feng, Xutong Ding, Ying Li, Huiyuan Fu, Shiquan Liang, Qili Yao, Zhenfeng Zhang, Feng Li, Stephan Urban, Hongbo Guo, Wenshi Wang

**Affiliations:** 1 Department of Pathogen Biology and Immunology, Jiangsu Key Laboratory of Immunity and Metabolism, Jiangsu International Laboratory of Immunity and Metabolism, Xuzhou Medical University 666893 , Xuzhou, China; 2 Institute of Infectious Diseases, Guangzhou Eighth People’s Hospital, Guangzhou Medical University 26468, Guangzhou, China; 3 School of Public Health and Emergency Management, Southern University of Science and Technology 255310, Shenzhen, China; 4 Scientific Research Center, Shanghai Public Health Clinical Center, Fudan University 34748, Shanghai, China; 5 Department of Infectious Diseases, Molecular Virology, University Hospital Heidelberg 64338, Heidelberg, Germany; 6 German Centre for Infection Research (DZIF) Partner Site Heidelberg, Heidelberg, Germany; Wake Forest University School of Medicine, Winston-Salem, North Carolina, USA

**Keywords:** HDV genotypes, interferon-alpha, cell division-mediated spread, *de novo* infection, resting cells

## Abstract

Chronic hepatitis delta virus (HDV) infection causes the most severe form of viral hepatitis. Although humans produce 12 subtypes of interferon-alpha (IFN-α), IFN-α2a has been the only commonly used treatment against HDV. Previously, we characterized eight HDV genotype isolates with varying replication kinetics. Herein, we systematically investigated the antiviral efficacy of IFN-α2a and other IFN-α subtypes against HDV genotypes 1–8 during *de novo* infection, cell mitosis, and in quiescent cells. Our findings revealed that IFN-α2a exhibits potent but varied efficiency against HDV 1–8 isolates upon *de novo* infection and cell mitosis. Conversely, HDVs in resting cells are resistant to IFN-α2a treatment and the IFN-containing cytokine cocktail collected from peripheral blood mononuclear cells stimulated with TLR7/8 agonist. Mechanistically, both ADAR1 p110 and p150 promote L-HDAg production and inhibit HDV replication. ADAR1 p150, rather than p110, enhances the anti-HDV efficacy of IFN-α during *de novo* infection and cell mitosis, but not in resting cells. Moreover, different subtypes of IFN-α exhibit varying anti-HDV activities in both *de novo* infection and cell mitosis, due to their disparity in activating interferon responses. Among these, IFN-α2a, IFN-α10, and IFN-α14 exhibit the strongest anti-HDV activity and synergize with bulevirtide in suppressing HDV replication. In conclusion, the anti-HDV efficacy of IFN-α depends on multiple factors, including HDV genotypes, ADAR1 p150 level, IFN-α subtypes, and HDV’s different survival strategies. These findings provide valuable implications for the development and optimization of IFN-based therapies.

**IMPORTANCE:**

Chronic hepatitis delta virus (HDV) infection represents the most severe form of viral hepatitis. This study comprehensively evaluated the antiviral efficacy of interferon-alpha (IFN-α) subtypes across eight HDV genotypes during *de novo* infection, cell mitosis, or in quiescent cells. Herein, we found that IFN-α exhibits potent but varied efficiency against HDV 1–8 isolates upon *de novo* infection and cell mitosis. Conversely, HDVs in resting cells are resistant to IFN-α subtypes, regardless of the cellular ADAR1 levels. Among different subtypes, IFN-α2a, IFN-α10, and IFN-α14 exhibit the strongest anti-HDV activity and synergize with bulevirtide in suppressing HDV replication. These findings provide crucial insights into the optimization of IFN-based monotherapy and combinational therapy against chronic HDV infection.

## INTRODUCTION

As a satellite virus of hepatitis B virus (HBV), hepatitis delta virus (HDV) is considered the human pathogen with the smallest known genome (~1.7 kb). HDV and HBV co-infection causes the most severe form of chronic viral hepatitis and significantly accelerates the progression of liver fibrosis, cirrhosis, and hepatocellular carcinoma (1). Despite the promotion of the HBV vaccination program, recent studies indicate that the prevalence of HDV has either remained stable or increased globally (2–5). It is estimated that 12–72 million people are chronically infected with HDV, causing substantial global morbidity and mortality (2–5).

HDV virion comprises a ribonucleoprotein (RNP) complex and an HBV envelope. Upon *de novo* infection, HDV RNP was released in the cytoplasm and further translocated to the nucleus to initiate viral replication (6–8). In the nucleus, the genomic RNA serves as the template for the synthesis of the HDV mRNAs, allowing the production of S-HDAg. During replication, antigenomic RNA is edited by host adenosine deaminase acting on RNA 1 (ADAR1), introducing an A/G mutation in the amber stop codon of the S-HDAg open reading frame. Consequently, a second mRNA is produced coding for the L-HDAg with a C-terminal extension of 19 or 20 amino acids. In contrast to S-HDAg, required to initiate and promote HDV replication, the C-terminal extension confers L-HDAg dual distinct functions: inhibiting HDV RNA replication and supporting virion production (9). Besides HBV envelope-dependent *de novo* infection, recent studies indicated that HBV-independent cell division-mediated spread and long-lasting viral replication in resting cells collectively contribute to HDV persistence (10–13).

The exploration of HDV virology has led to the development of novel therapeutic agents (14). For instance, the entry inhibitor bulevirtide (Hepcludex/Myrcludex-B, BLV) received conditional approval from the European Medicines Agency in 2020 as the first HDV-specific drug (15), and the farnesyl transferase inhibitor lonafarnib is being tested in clinical trials. Besides these advances, off-label interferon-alpha (IFN-α) (subtype 2a) has been the only commonly used treatment against HDV for decades (16). IFN-α2a or peg-IFN-α2a monotherapy is only partially effective and not curative for most chronic hepatitis D patients (17, 18). Nevertheless, recent clinical studies have demonstrated that combinations of IFN-α with BLV and drugs under development (e.g., lonafarnib) exhibited additive or even strong synergistic antiviral effects in terms of faster and more profound reductions in serum HDV RNA, higher off-treatment responses, and lower relapse rates (11, 19–28). These observations highlight the essential role of IFN-α in combating HDV infection, whether through IFN monotherapy or IFN-based combination therapies.

HDV is composed of eight genotypes (HDV-1 to HDV-8) defined by an intergenotype similarity >85% or >80%, according to the partial or full-length genome sequence, respectively (29). Eight HDV genotypes exhibited a specific phylogeographic distribution pattern worldwide (HDV-1: worldwide distribution; HDV-2: Southeast and East Asia; HDV-3: South America; HDV-4: Japan and China; and HDV-5 to HDV-8: Africa) (3, 29). Previously, we cloned and characterized eight HDV 1–8 isolates, exhibiting marked differences in replication efficacy and virus production kinetics (30). Intriguingly, following IFN-α2a treatment, varied treatment responsiveness and prognoses have been reported among patients infected with different HDV genotypes. For instance, a London retrospective study found that patients infected with HDV-5 appeared to have a better prognosis with fewer episodes of hepatic decompensation and better response to peg-IFN treatment than patients infected with HDV-1 (31). In addition, a single Brazilian study reported an unusually high response rate (>95%) to a peg-IFN-entecavir combination treatment in non-European HDV-3-infected patients, suggesting that HDV-3 might represent an “easy-to-treat” variant compared to HDV-1 (32). These clinical discoveries underscore the importance of conducting the comparative investigation of IFN-α against HDV 1–8 isolates.

The cellular innate immune system plays a crucial role in virus suppression through the initiation of direct antiviral responses. As the most well-known antiviral cytokine, IFN-α induces hundreds of interferon-stimulated genes (ISGs) via the Janus kinase signal transducer and activator of transcription (JAK-STAT) signaling pathway, thereby triggering an active cellular antiviral state (33). Interestingly, humans express 12 IFN-α subtypes that share 75%–99% amino acid sequence identity (34, 35). Although they bind to the same cellular receptor IFNAR1/IFNAR2 and eventually induce ISG expression via the JAK-STAT pathway, different subtypes exhibit distinct antiviral capabilities against different viruses. For instance, IFN-α14 exhibited the strongest antiviral activity against both HBV and HIV-1 (36, 37), whereas IFN-α5 was superior against severe acute respiratory syndrome coronavirus 2 infection (38). Moreover, IFN-α16, IFN-α5, and IFN-α4 suppressed influenza A H3N2 replication with up to 230-fold greater efficiency than IFN-α2 (39). However, IFN-α2 outperformed other subtypes against the hepatitis E virus (40). To date, only the subtype IFN-α2 is widely used for HDV treatment. The therapeutic potential of other subtypes remains largely unknown.

Herein, we systematically analyzed the anti-HDV potency of IFN-α2a against HDV 1–8 isolates across three distinct conditions: (i) HDV *de novo* infection, (ii) HDV replication in resting cells, and (iii) HDV transmission in dividing cells. Moreover, a mixture of IFN-containing cytokines was obtained from peripheral blood mononuclear cells (PBMCs) stimulated with TLR7/8 agonist, and its effectiveness against HDV was subsequently evaluated in these three different HDV surviving conditions. To investigate mechanisms influencing IFN-α efficacy, we explored the role of the host enzyme ADAR1 in modulating the antiviral response. Furthermore, the anti-HDV capabilities of different IFN-α subtypes were compared in three conditions as well. The underlying mechanisms that contribute to the anti-HDV disparity of IFN-α subtypes were elucidated accordingly. Moreover, the antiviral effect of the most potent IFN-α subtypes in combination with BLV was evaluated. This multimodal characterization of the antiviral effects of IFN-α subtypes against HDV 1–8 offers valuable insights for the development and optimization of IFN monotherapy and combinational therapies.

## MATERIALS AND METHODS

### Plasmids

Plasmids pcDNA3.1-HDV-1 to pcDNA3.1-HDV-8 containing 1.1 copy antigenome of HDV genotypes 1–8 (Table S1) and pLX304-HB2.7-B (NCBI accession number: MN645904) were generated as described previously (30). The pcDNA3.1-G418-HDV-1 to pcDNA3.1-G418-HDV-8 plasmids were generated by inserting the 1.1 copy antigenome of HDV genotypes 1–8 into pcDNA3.1 (+) vector harboring the neomycin resistance gene, respectively. The plasmid pJC126 (9, 41) harbored a 1.1-fold copy of the HDV (genotype 1) antigenome and a neomycin resistance gene, designated as pcDNA3.1-G418-HDV-1T. Plasmids pWPI-puro-ADAR1 p150 and pWPI-puro-ADAR1 p110 were generated by inserting human double-stranded RNA adenosine deaminase 1 (ADAR1) p150 (NCBI accession number: U10439) or spliced p110 into the lentiviral pWPI vector. For knocking down ADAR1, short-hairpin RNAs (shRNAs) were designed and inserted into the lentiviral pLVX vector (pLVX-puro-shADAR1). We also designed and cloned the sgRNA sequences into the pLentiCRISPR v.2 vector (Addgene vector #52961) for the knockout of the ADAR1 gene by the CRISPR/Cas9 system.

### Cells

Human NTCP overexpressing HepG2^NTCP^ and HuH7^NTCP^ cells were generated as described previously (30, 42–44). HepaRG^NTCP^ cells were kept in AG Urban’s lab (University Hospital Heidelberg). For the generation of HuH7-HDV-1T and HuH7-HDV-1 to -HDV-8 cells, HuH7 cells were transfected with pcDNA3.1-G418-HDV-1T and pcDNA3.1-G418-HDV-1 to pcDNA3.1-G418-HDV-8 plasmids, respectively. Positive cells were selected with G418. For the generation of ADAR1 shRNA knockdown or CRISPR knockout cells (shADAR1 or CRISPR-ADAR1 cells), HuH7^NTCP^ cells were transduced with lentiviruses encoding shRNA or sgRNA or their respective controls (shCTR and CRISPR-CTR), followed by puromycin selection. For the generation of ADAR1 overexpression cells, HuH7^NTCP^ cells were transduced with lentiviruses encoding ADAR1 p150 or ADAR1 p110 (OE-ADAR1 p150 or OE-ADAR1 p110) or overexpression control (OE-CTR) and followed by puromycin selection. The technical support for preparing the primary hepatocytes/non-parenchymal cells was provided by Liver Biotech (Shenzhen, China).

### Virus production and infection

The HDV virus particles (HDV-1T and HDV-1 to HDV-8) were produced by co-transfection of HuH7 cells with the corresponding HDV plasmids (pJC126, pcDNA3.1-HDV-1 to pcDNA3.1-HDV-8) and pLX304-HB2.7-B as previously described (9, 30). For infection assays, hepatoma cell lines and primary human hepatocyte (PHH) cells were inoculated with 1/5 to 2/5 of the cell culture supernatant (if not mentioned otherwise) in a medium containing 4% polyethylene glycol (PEG) and 1.5% dimethyl sulfoxide (DMSO). After 16 hours of inoculation, the cells were washed twice with phosphate-buffered saline (PBS) or PHH maintenance medium, and the fresh DMEM medium or PHH maintenance medium containing 1.5% DMSO was provided and replaced every second day until the end of the experiment. For HDV cell division spreading assays, HDV-infected cells were trypsinized at 5-day post-infection (p.i.) and passaged at the 1:12 dilution to undergo cell division-mediated HDV spread (10). The HBV virus particles were produced by collecting the supernatant of the HepAD38 cells as previously described (45).

### *De novo* infection, dividing-cell (spread) model, and resting-cell model

HDV *de novo* infection and HDV spread in dividing cells and resting cells were performed as previously described (10, 46).

#### *De novo* infection

Cells were seeded to ∼100% confluence and maintained in 1.5% DMSO-containing medium. The next day, cells were inoculated with HDV for 16 hours, then washed and treated with IFN-α2a. On day 5 post-infection (when HDV replication was fully established) (10, 46), samples were harvested to assess the inhibitory effect of IFN on HDV (10, 46).

#### Dividing-cell (spread) model and resting-cell model

Cells were seeded to ∼100% confluence and maintained in 1.5% DMSO-containing medium. On day 5 post-infection (when HDV replication was fully established) (10, 46), cells were passaged at a 1:12 split and treated with IFN-α2a from day 5 to day 9. This condition allows quantification of IFN-α2a’s impact on HDV propagation through cell division (10, 46). This is the so-called dividing-cell (spread) model. In parallel, on day 5 post-infection, cells were kept in 1.5% DMSO-containing medium without passaging. Under these non-dividing conditions, HDV replication occurs without new rounds of *de novo* infection and cell division-mediated spread (10, 46). This is the so-called resting-cell model.

### Preparation of recombinant IFN-I subtypes

The human IFN-α2a was purchased from PBL Assay Science (11101-1). Thirteen recombinant human IFN-I subtypes were produced as previously described (36, 37, 47). The human IgG1-linked type I interferon gene was inserted into the corresponding expression vector pCMV3. For transient transfection, plasmids were combined with sinofection transfection reagent (Catalog #STF02, Sinobiological) and introduced into HEK293 cells cultured in a serum-free medium. The cells were maintained in Erlenmeyer flasks on an orbital shaker or in a bioreactor with appropriate stirring at 37°C for 6 days. Subsequently, cell culture supernatants were collected and processed using affinity purification columns. The purified proteins were then analyzed via SDS-PAGE and quantified by UV–visible spectrophotometer.

### Production of IFN-containing cytokine mixture

The PBMCs were isolated from whole blood by Ficoll-Paque PLUS density gradient media (GE, 17-1440-02) under the guidance of the Ethical Committee. The TLR7/8 agonist resiquimod (R848) was purchased from Selleck (S8133) and diluted with DMSO to a final stock concentration of 10 mM. PBMCs were supplemented with culture medium (RPMI1640, 10% fetal bovine serum [FBS]) with 200 nM R848 or DMSO. The cell culture supernatants of PBMCs were collected after 24 hours post-incubation with R848 or DMSO.

### Indirect immunofluorescence

Immunofluorescence (IF) was performed as previously described (9, 30). In short, cells were fixed with 4% paraformaldehyde (PFA) for 15 minutes at room temperature, followed by 30 minutes of incubation in permeabilization buffer (PBS, 0.25% Triton X-100). Then, cells were incubated with rabbit anti-HDAg polyclonal antibody diluted in 2% bovine serum albumin (BSA, 1:4,000, 4°C, 12 hours). CoraLite 594-conjugated goat anti-rabbit secondary antibody (Proteintech, SA00013-4, 1:500, 37°C, 1 hour) and Hoechst 33342 dye were used. Images were taken with an Olympus IX51 inverted microscope, and image analysis and quantification were performed using ImageJ software.

### RT-qPCR

Reverse transcription quantitative PCR (RT-qPCR) was performed as previously described (9, 30). Briefly, total RNA was extracted from cells using the TRIzol RNA extraction kit (Invitrogen, 15596026) according to the protocol provided. The RNA secondary structure was melted by incubation of RNA at 95°C for 5 minutes, followed by immediate cooling down to −80°C. Then RNA was reverse transcribed using the HyperScript First-Strand cDNA Synthesis kit (APExBIO, K1071). Quantitative HDV RNA was performed on a Roche LightCycler 480 system using Luna Universal Probe qPCR Master Mix (New England Biolabs, M3004X) with the primers depicted in Table S2. SYBR Green Supermix (APExBIO, K1070) was used for detecting ISGs mRNA, HBV total RNA, and HBV pregenomic RNA with primers detailed in Table S2.

### In-cell enzyme-linked immunosorbent assay

In-cell enzyme-linked immunosorbent assay was performed as previously described (9, 30). Cells were cultured in white, non-transparent 96-well plates and fixed in 4% PFA for 30 minutes, followed by 30 minutes of incubation in permeabilization buffer (PBS, 0.25% Triton X-100). After 30 minutes of incubation in blocking buffer (PBS, 0.05% Tween-20, 3% BSA), cells were incubated for 2 hours with the anti-HDAg monoclonal antibody. After extensive washing, endogenous peroxidases were blocked with a 3% hydrogen peroxide solution (10 minutes). Cells were incubated with a secondary goat anti-rabbit HRP (Jackson Immuno Research) antibody for 1 hour. After extensive washing, the chemiluminescence substrate (Advansta ELISABright) was added to the wells, and luminescence was measured on a plate reader.

### Western blot

Cell lysates were applied to SDS-PAGE on 10% SDS gels. Proteins were transferred onto nitrocellulose membranes by semidry transfer and incubated with primary antibodies as indicated below: rabbit anti-HDAg polyclonal antibody, mouse anti-β-actin (Sigma, A1978), mouse anti-GAPDH (Proteintech, 60004-1-lg), rabbit anti-ADAR1 (Cell Signaling, 14175), STAT1 monoclonal antibody (Proteintech, 66545-1-lg), STAT2 polyclonal antibody (Proteintech, 16674-1-AP), p-STAT1: phospho-STAT1 (Tyr701) polyclonal antibody (Proteintech, 28979-1-AP), and phospho-STAT2 (Tyr690) antibody (Affinity, AF3342). After overnight incubation at 4°C, membranes were washed with Tris-buffered saline with Tween-20 (TBST), and incubated with fluorescent-labeled secondary antibodies (LI-COR Biosciences) for 1 hour at room temperature, washed again, and imaged on a LI-COR Odyssey imaging system.

### Cell viability assay

WST-1 reagent from the cell proliferation and viability detection kit (KeyGen Biotech, KGA316) was added to the cells in a 96-well plate. Cells were maintained at 37°C with 5% CO_2_ for 2 hours. The absorbance of each well was read on the microplate absorbance readers (Bio-Rad, USA) at the wavelength of 450 nm.

### Interferon-stimulated response element luciferase reporter assays

HuH7^NTCP^ and HepG2^NTCP^ cells were transiently transfected with pGL4.45-luc2P-ISRE-Hygro and pRL-TK (a plasmid expressing the Renilla luciferase protein was used as an internal control) plasmids in 96-well cell culture plates. Eight hours post-treatment with IFN-α, the luciferase values were measured using the duo-lite luciferase reporter assay system (Vazyme, DL101-01) according to the manufacturer’s instructions. Data were normalized by determining the ratios of firefly luciferase activities to that of Renilla luciferase (48).

### Statistical analysis

Statistical significance between two groups was determined using unpaired two-tailed Student’s *t*-test, and one-way ANOVA analysis was used to compare the means of three or more groups in the software GraphPad Prism 9 (*P  <* *0.05, **P <* *0.01, ***P <* *0.001, ****P <* *0.0001, NS: not significant, *P* > 0.05). All error bars throughout the study represent the standard error of the mean.

## RESULTS

### IFN-α2a exhibited potent but varied antiviral efficacy against HDV 1–8 isolates upon *de novo* infection

**Fig 1:**
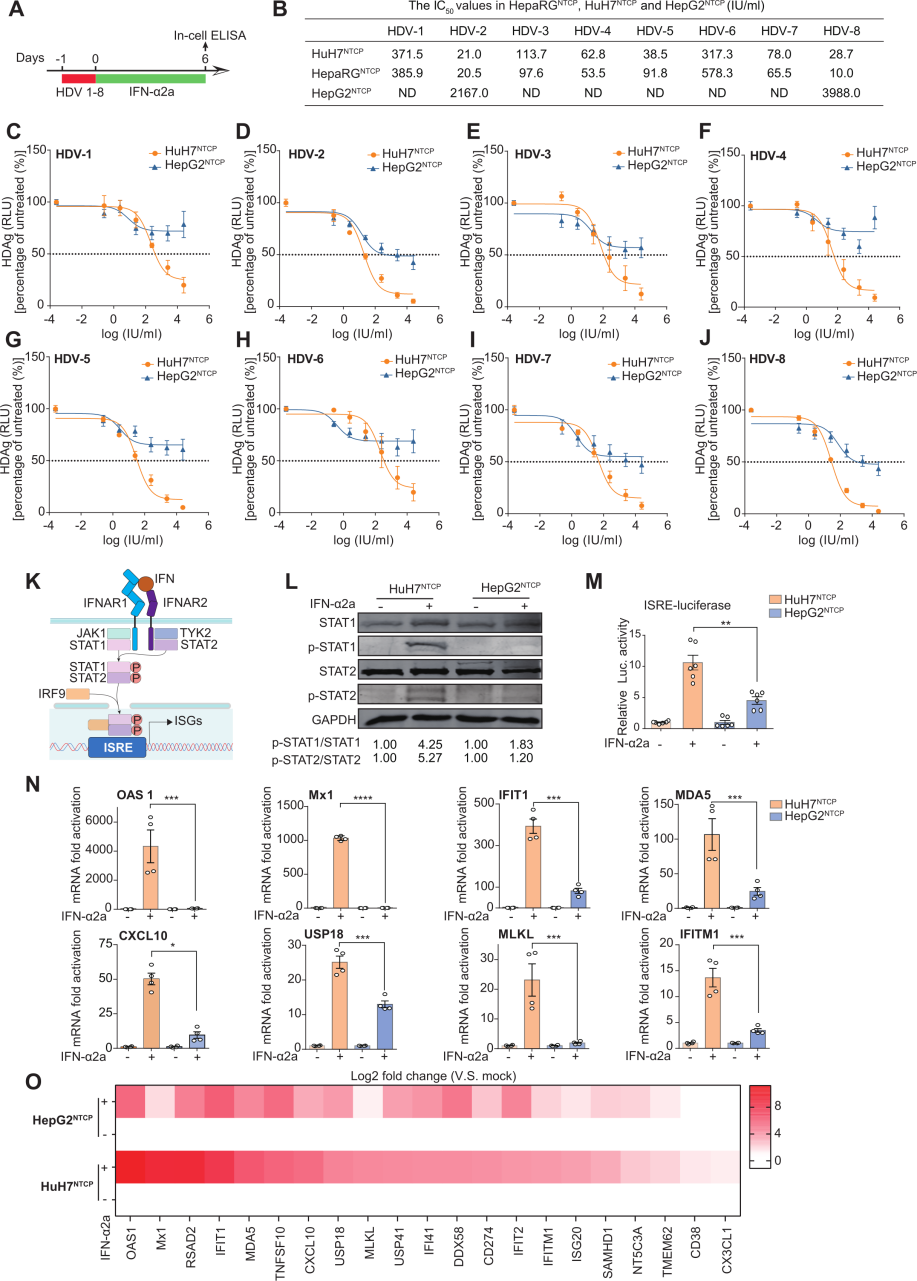
Characterization of the antiviral efficacy of IFN-α2a against HDV 1–8 isolates upon HDV *de novo* infection. (**A**) Schematic of the experimental setting. (**B**) The IC_50_ values of IFN-α2a against HDV 1–8 isolates in HuH7^NTCP^, HepaRG^NTCP^, and HepG2^NTCP^ cells. (**C–J**) The antiviral efficacy of IFN-α2a against HDV 1–8 isolates was tested in HuH7^NTCP^ and HepG2^NTCP^ cells upon HDV *de novo* infection (*n* = 4). (**K**) Schematic model of JAK-STAT signaling pathways. (**L**) HuH7^NTCP^ and HepG2^NTCP^ cells were treated with IFN-α2a (1,000 IU/mL) for 30 minutes. The levels of STAT1, STAT2, pSTAT1(Y701), and pSTAT2(Y690) were detected by western blot. The ratios of p-STAT1/STAT1 or p-STAT2/STAT2 were calculated by normalizing IFN-α2a-induced p-STAT1 or p-STAT2 level to both GAPDH and the baseline STAT1 or STAT2 expression. (**M**) HuH7^NTCP^ and HepG2^NTCP^ cells were treated with IFN-α2a (1,000 IU/mL), and the levels of interferon-stimulated response element (ISRE)-related luciferase values were detected 8 hours post-treatment (*n* = 6). (**N and O**) HuH7^NTCP^ and HepG2^NTCP^ cells were treated with IFN-α2a (1,000 IU/mL) for 8 hours. A of representative ISGs was analyzed by RT-qPCR (*n* = 4). The data were presented either in bar chart (**N**) or heatmap (**O**). HDAg, hepatitis D antigen; RLU, relative light unit; IF, immunofluorescence; Luc, luciferase; ND, not detected. *P  <* *0.05, **P <* *0.01, ***P <* *0.001, ****P <* *0.0001.

Patients infected with different HDV genotypes showed varied treatment responsiveness and prognoses to IFN-α treatment (31, 32, 49). Given that multiple factors, including HDV genotypes and individual differences of patients, may contribute to treatment response in clinical settings, studies on the role of HDV genotype in response to IFN-α using more simplified *in vitro* infection systems were optimal. Therefore, cell culture-derived patient HDV 1–8 isolates were generated *in vitro* as described previously (30). HuH7^NTCP^ cells were inoculated with HDV 1–8 and treated with different concentrations of IFN-α2a (Fig. 1A). As shown in Fig. 1B through J, IFN-α2a dose-dependently inhibited HDV 1–8 upon *de novo* infection, with no cytotoxicity (Fig. S1A). Specifically, the IC_50_ values varied in HuH7^NTCP^ cells (IC_50_: HDV-2, HDV-8 < HDV-5, HDV-4, HDV-7, HDV-3 < HDV-6, HDV-1), whereas different HDV infection rates exerted no significant effect on IC_50_ values (Fig. S2). Consistently, similar genotype-specific responsiveness was observed in HepaRG^NTCP^ (IC_50_: HDV-8, HDV-2 < HDV-4, HDV-7, HDV-5, HDV-3 < HDV-1, HDV-6, Fig. 1B; Fig. S3), further highlighting the genotype-specific susceptiveness of HDV to IFN-α2a.

IFN-α exerted its cellular antiviral activity by binding to the cellular receptor IFNAR1 and IFNAR2 to activate the JAK-STAT pathway, thus inducing hundreds of ISGs (Fig. 1K). Interestingly, despite similar expression levels of IFNAR1 and IFNAR2 in HuH7^NTCP^ and HepG2^NTCP^ cells (Fig. S4), IFN-α2a showed a weakened antiviral effect against HDV 1–8 in HepG2^NTCP^ cells (Fig. 1B through J), with no cytotoxicity (Fig. S1B). Notably, IFN-α2a induced higher levels of phosphorylated STAT1 and STAT2 in HuH7^NTCP^ cells compared to HepG2^NTCP^ cells (Fig. 1L). Consistently, in comparison with HepG2^NTCP^ cells, IFN-α2a treatment elicited stronger transactivation of interferon-stimulated response elements (ISREs) driven luciferase activity in HuH7^NTCP^ cells (Fig. 1M). Moreover, IFN-α2a provoked higher levels of the most well-studied antiviral ISGs in HuH7^NTCP^ cells (Fig. 1N and O; Fig. S5). These data clarified the differences in how IFN-α2a functioned in different cell models, indicating that the cell’s intrinsic IFN response played a crucial role in determining the anti-HDV efficacy of IFN-α. Collectively, both HDV genotypes and the cell-intrinsic IFN response contributed to the differential sensitivity to IFN-α treatment during *de novo* HDV infection.

### In contrast to the minor anti-HDV effect in resting cells, IFN-α2a manifested efficient but varying antiviral potency against HDV 1–8 isolates during cell division

**Fig 2:**
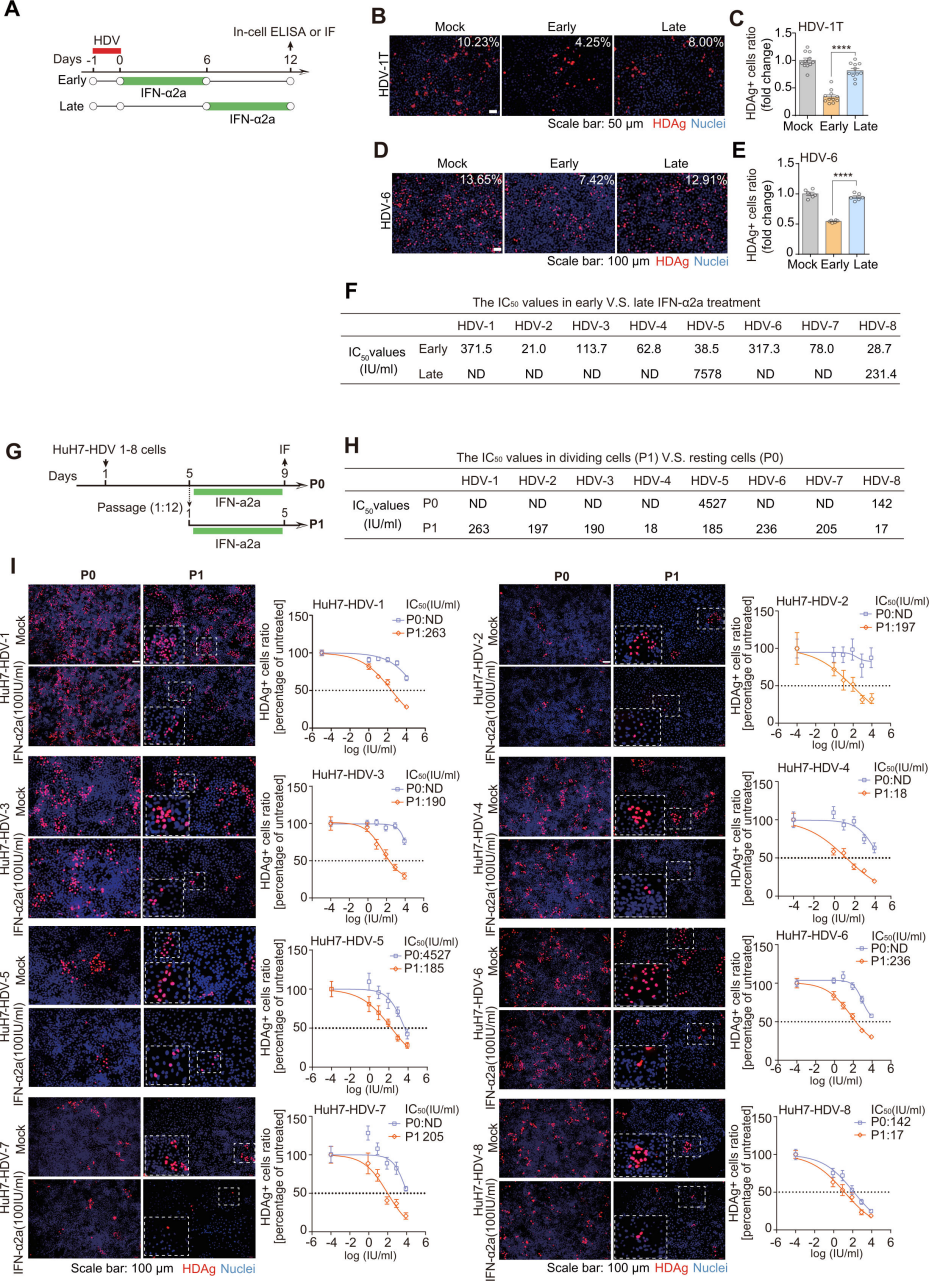
Characterization of the antiviral efficacy of IFN-α2a against HDV 1–8 isolates in resting or dividing cells. (**A**) Schematic of the experimental setting. (**B and C**) HDV-1T-infected HuH7^NTCP^ cells were treated with IFN-α2a (1,000 IU/mL) from day 0 to day 6 (early) or from day 6 to day 12 (late). HDAg-positive cells were quantified at day 12 post-infection (*n* = 6, ****P <* *0.0001). (**D and E**) Same as (**B and C**) for HDV-6 (****P <* *0.0001). (**F**) The IC_50_ values in early versus late IFN-α2a treatment groups were quantified. (**G**) Schematic of the experimental setting. HuH7-HDV-1 to HuH7-HDV-8 cells were either passaged at a 1:12 dilution (P1, dividing cells) or left without passaging (P0, resting cells). Next, cells were treated with different doses of IFN-α2a for 5 days. (**H**) The IC_50_ values of IFN-α2a against HDV 1–8 isolates in P0 and P1 groups were quantified. (**I**) The relative ratios of HDAg-positive cells were quantified 5 days post-IFN treatment (*n* = 4).

Besides *de novo* infection, HDV employed two additional strategies to ensure its persistence: cell division-mediated HDV spread and the maintenance of HDV in resting cells (11). Previously, based on the HDV-1 derivative (HDV-1T, pJC126), it was reported that in contrast to *de novo* infection, HDV replication was insensitive to IFN treatment once the infection was established (at days 5–6 p.i.) (46). Consistently, our indirect IF assay showed that IFN-α2a significantly inhibited HDV-1T and HDV-6 in the early treatment group (treated with IFN-α2a upon HDV *de novo* infection, days 0–6), whereas its antiviral effect was significantly diminished in the late treatment group (treated with IFN-α2a after the establishment of infection, days 6–12) (Fig. 2A through E). This observation was further confirmed in the HBV-HDV co-infection model (Fig. S6). These data prompted us to investigate whether such resistance is a pan-genotypic characteristic of HDV. Indeed, although IFN-α2a elicited comparable ISG levels in early and late treatment groups (Fig. S7), it exhibited a limited antiviral activity against HDV 1–8 isolates in the late treatment group, contrasting with the robust and dose-dependent effects observed in the early treatment group (Fig. 2F; Fig. S8). Interestingly, HDV-5 and HDV-8 isolates exhibited relatively better responsiveness in the late treatment group compared to other genotypes (Fig. 2F; Fig. S8).

Next, based on HuH7 cells, HuH7-HDV-1 to HuH7-HDV-8 cells were generated as described previously (9, 50). These eight cell lines continually supported HDV replication of HDV 1–8 isolates, whereas no *de novo* HDV infection occurred due to the lack of NTCP and HBV envelope proteins. Thus, these eight cell lines represented an optimal *in vitro* model to investigate the responsiveness of IFN-α against HDV 1–8 in cell resting or dividing state (Fig. 2G). According to previous studies, we chose day 5 p.i. in time for the passage of HDV-positive cells (10, 46). Consistent with the late treatment group in Fig. 2F, HDV 1–8 isolates were refractory to IFN-α2a treatment in the cell resting state (P0), albeit with HDV-5 and HDV-8 showing relatively better response compared to other genotypes (Fig. 2H and I). Conversely, IFN-α2a exhibited a much stronger and dose-dependent antiviral effect against cell division-mediated HDV 1–8 spread (P1), with IC_50_ values varying among different genotypes (IC_50_: HDV-8, HDV-4 < HDV-5, HDV-3, HDV-2, HDV-7, < HDV-6, HDV-1) (Fig. 2H and I). In sum, compared with the limited anti-HDV effect in resting cells, IFN-α2a potently inhibited HDV 1–8 in *de novo* infected and propagated via cell division, with efficacy varying among different genotypes.

### HDV in resting cells was refractory even with the treatment of IFN-containing cytokine cocktails

**Fig 3:**
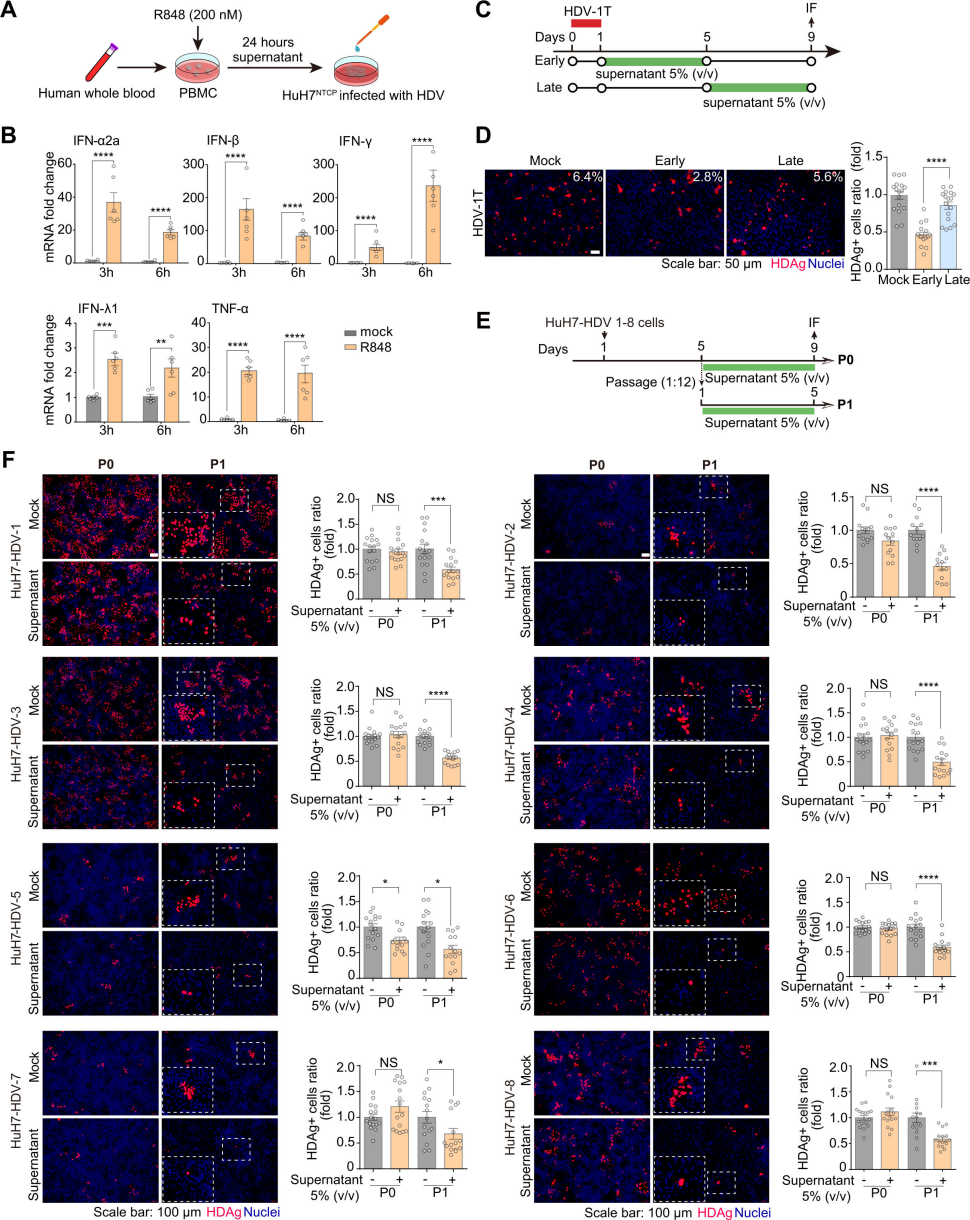
The antiviral efficacy of IFN-containing cytokine cocktail collected from PBMCs stimulated with TLR7/8 agonist. (**A**) Schematic of the protocol to produce IFN-containing cytokine cocktail with PBMCs. (**B**) The total RNA of human PBMCs was extracted after 3 hours or 6 hours post-incubation with TLR7/8 agonist (R848, 200 nM). The mRNA levels of IFN-I (α2a, β), IFN-II (γ), IFN-III (λ1), and TNF-α were quantified by RT-qPCR (*n* = 3). (**C**) Schematic of the experimental setting. (**D**) HuH7^NTCP^ cells were infected with HDV-1T. The anti-HDV efficacy of IFN-containing cytokine cocktail (supernatant, 5%, vol/vol) was detected by IF in the setting of both early treatment and late treatment (*n* = 6). (**E**) Schematic of the experimental setting. (**F**) HuH7-HDV-1 to HuH7-HDV-8 cells were either passaged at a 1:12 dilution (P1, dividing cells) or left without passaging (P0, resting cells). Next, cells were treated with IFN-containing cytokine cocktail (supernatant, 5%, vol/vol) for 5 days. HDAg-positive cells were quantified at day 5 post-treatment (*n* = 4). *P  <* *0.05, **P <* *0.01, ***P <* *0.001, ****P <* *0.0001, NS: not significant, *P* > 0.05.

Currently, TLR7/8 agonists are in clinical development for the treatment of chronic hepatitis B (51–53). Mechanistically, TLR7/8 agonists potently activate PBMCs to promote the release of a mixture of cytokines (e.g., tumor necrosis factor-α [TNF-α] and IFNs) to exert a collective antiviral effect (54–56). Herein, TLR-7/8 agonist resiquimod (R848) was used to investigate whether an IFN-containing cytokine cocktail can efficiently inhibit HDV, especially in resting cells. This condition was more biologically relevant, as cells *in vivo* usually encounter multi-cytokines, rather than one cytokine alone. Thus, we stimulated PBMCs with R848 and harvested the supernatant (Fig. 3A), which included multiple cytokines, e.g., IFN-α2a, IFN-β, IFN-γ, IFN-λ1 and TNF-α (Fig. 3B; Fig. S9). Next, we evaluated the anti-HDV effect of the cytokine cocktail in three different conditions: *de novo* infection (Fig. 3C and D, early), HDV in resting cells (Fig. 3C and D, late, and Fig. 3E and F, P0), and cell division (Fig. 3E and F, P1). Similar to IFN-α2a treatment, R848-induced cytokine cocktail significantly inhibited HDV upon *de novo* infection or cell mitosis, whereas no effect in resting cells (Fig. 3D and F). Interestingly, although HDV-5 and HDV-8 showed a relatively better response with IFN-α2a treatment (P0), their response to R848-stimulated PBMCs cocktail (P0) was much weaker (Fig. 2I and 3F). This was probably due to the fact that the cytokine composition and concentrations were not comparable in these two different settings. In sum, these results indicated that HDV in resting cells exhibited strong resistance with the presence of a mixture of IFN-containing cytokines.

### IFN-inducible ADAR1 p150 contributed to the anti-HDV efficacy of IFN-α during HDV de *novo* infection and cell mitosis, rather than in resting cells

The ADAR1 gene generated two isoforms via alternative splicing events: the full-length p150 and the spliced p110 (57). IFN induced the expression of full-length p150 (Fig. S10A and B) that could efficiently edit the amber/W site (58). HDV replication was sensitive to ADAR1 editing levels due to the determination of L-HDAg/S-HDAg ratios (57, 59). Both shRNA and CRISPR/Cas9 methods were used to inhibit the expression of both forms of ADAR1 (p110 and p150) (Fig. S10C and G). As expected, the ratios of L-HDAg/S-HDAg in shADAR1 and CRISPR/Cas9-ADAR1 samples decreased dramatically compared to their control samples (Fig. S10C and G). For instance, at day 6, compared with shCTR and CRISPR-CTR cells, the L/S ratios in shADAR1 and CRISPR-ADAR1 cells decreased by 67% (Fig. S10C) and 93% (Fig. S10G), respectively. Notably, both the shADAR1 and CRISPR/Cas9-ADAR1 samples showed significantly increased levels of HDV replication (Fig. S10D through F and H through J). Conversely, overexpression of p150 or p110 increased the ratios of L-HDAg/S-HDAg and inhibited HDV replication during *de novo* infection (Fig. S10K through N). For instance, at day 6, compared with OE-CTR cells, the L/S ratios in OE-ADAR1 p150 and OE-ADAR1 p110 cells increased 4.23-fold (Fig. S10K) and 3.65-fold (Fig. S10M), respectively. Hence, both p150 and p110 edit the amber/W site to facilitate the production of L-HDAg, thus increasing the ratio of L-HDAg/S-HDAg and inhibiting HDV replication in consequence.

**Fig 4:**
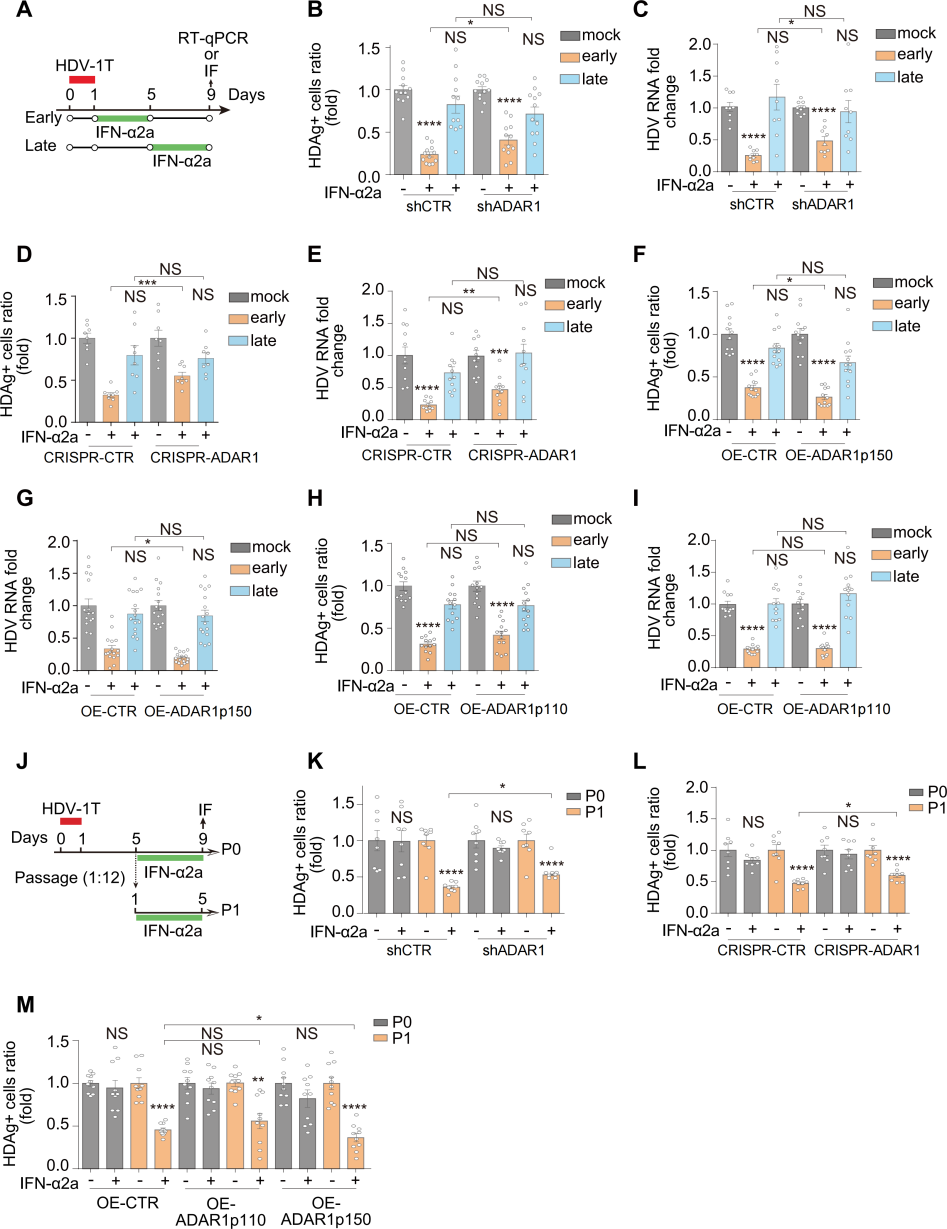
The roles of ADAR1 p110 and p150 on the anti-HDV efficacy of IFN-α2a. (**A**) Schematic of the experimental setting. (**B**) The shCTR and shADAR1 cells were infected with HDV-1T and then treated with IFN-α2a (1,000 IU/mL) from day 1 to day 5 (early) or from day 5 to day 9 (late). HDAg-positive cells were imaged and quantified at day 9 post-infection (*n* = 6). (**C**) The relative levels of intracellular HDV RNA were quantified by RT-qPCR at day 9 post-infection (*n* = 6). (**D and E**) Same as (**B and C**) for CRISPR-CTR and CRISPR-ADAR1 cells. (**F and G**) Same as (**B and C**) for OE-CTR and OE-ADAR1 p150 cells. (**H and I**) Same as (**B and C**) for OE-CTR and OE-ADAR1 p110 cells. (**J**) Schematic of the experimental setting. (**K**) The shCTR and shADAR1 cells were infected with HDV-1T. Cells were either passaged at a 1:12 dilution (P1, dividing cells) or left without passaging (P0, resting cells). HDAg-positive cells were imaged and quantified 5 days post-IFN treatment (1,000 IU/mL) by IF (*n* = 4). (**L**) Same as (**K**) for CRISPR-CTR and CRISPR-ADAR1 cells. (**M**) Same as (**K**) for OE-CTR, OE-ADAR1 p150, and OE-ADAR1 p110 cells. *P  <* *0.05, **P <* *0.01, ***P <* *0.001, ****P <* *0.0001, NS: not significant, *P* > 0.05.

This prompted us to investigate the possible roles of ADAR1 in the responsiveness of HDV to IFN-α treatment in three different settings. Therefore, shADAR1 cells, CRISPR/Cas9-ADAR1 cells (CRISPR-ADAR1), and their respective control cells were subjected to mock, early IFN-α2a treatment, or late IFN-α2a treatment (Fig. 4A). Overall, in both shADAR1 and control cells (shCTR), IFN-α2a significantly inhibited HDV in the early treatment group, whereas no effect was observed in the late treatment group (Fig. 4B and C; Fig. S11B). Consistently, similar results were observed in CRISPR-ADAR1 and control cells (Fig. 4D and E; Fig. S11C). Notably, ADAR1 knockdown decreased the anti-HDV effect of early IFN-α2a treatment compared with shCTR (Fig. 4B and C, columns 2 and 5; Fig. S11B), whereas no significant difference was observed in the late treatment groups (Fig. 4B and C, columns 3 and 6; Fig. S11B). This observation was further validated in CRISPR-CTR and CRISPR-ADAR1 cells by both IF and RT-qPCR assays (Fig. 4D and E; Fig. S11C). Conversely, in the setting of early IFN treatment, p150 overexpression (OE-ADAR1 p150) enhanced the anti-HDV effect of IFN-α2a (Fig. 4F and G, columns 2 and 5; Fig. S11D), whereas p110 overexpression (OE-ADAR1 p110) resulted in no significant differences (Fig. 4H and I, columns 2 and 5; Fig. S11E). Mechanistically, IFN-α2a treatment further upregulates the levels of ADAR1 p150 in OE-CTR (2.1-fold) cells, OE-ADAR1 p150 (2.6-fold) cells, and OE-ADAR1 p110 (2.3-fold) cells, while having no effect on ADAR1 p110 levels (Fig. S12). This observation supported that IFN-inducible ADAR1 p150 enhanced the anti-HDV effect during IFN-α2a treatment, whereas ADAR1 p110 did not.

Next, we evaluated the efficacy of IFN-α2a in blocking HDV spread via cell division in ADAR1-overexpressing or ADAR1-knockout cell lines, with parallel assessments of HDAg positivity rates and cluster sizes (Fig. 4J; Fig. S11F and S13A). Consistent with the early IFN-α2a treatment groups (Fig. 4B through E), the downregulation of ADAR1 by shRNA or CRISPR/Cas9 methods slightly but significantly inhibited the anti-HDV efficacy of IFN-α2a in cell-dividing state (P1) (Fig. 4K and L; Fig. S11G, H, S13B and C, P1). Nevertheless, HDV replication in the cell resting state (P0) remained insensitive to IFN-α2a regardless of the levels of p110 and p150 (Fig. 4K through M; Fig. S11G through I, P0). Conversely, the overexpression of p150, rather than p110, promoted the anti-HDV efficacy of IFN-α2a in P1 cells (Fig. 4M, columns 4, 8, and 12; Fig. S13D, columns 2, 4, and 6). To examine whether the varied antiviral efficacy of IFN-α2a across HDV 1-8 (Fig. 1 and 2) was due to the differential expression levels of ADAR1, ADAR1 was quantified post-IFN-α2a treatment with the presence of different HDV genotypes. However, IFN-α2a treatment induced comparable levels of ADAR1 p150 (p110 remained unchanged) across cells infected with different HDV genotypes (Fig. S14A through D). Moreover, no significant differences in ADAR1 p150 and p110 were observed among cells infected with HDV 1–8 (Fig. S14E through N), indicating that genotype-dependent IFN sensitivity was not explained by differential ADAR1 induction either. In conclusion, acting as an anti-HDV ISG, IFN-α-inducible ADAR1 p150 contributed to, but was not solely responsible for, IFN-α-mediated anti-HDV activity during *de novo* infection and cell division. However, in resting cells, its role in IFN-α-mediated anti-HDV activity was negligible.

### IFN-α subtypes exhibited dose-dependent and varied anti-HDV activities during *de novo* infection and cell mitosis but were not effective in resting cells

**Fig 5:**
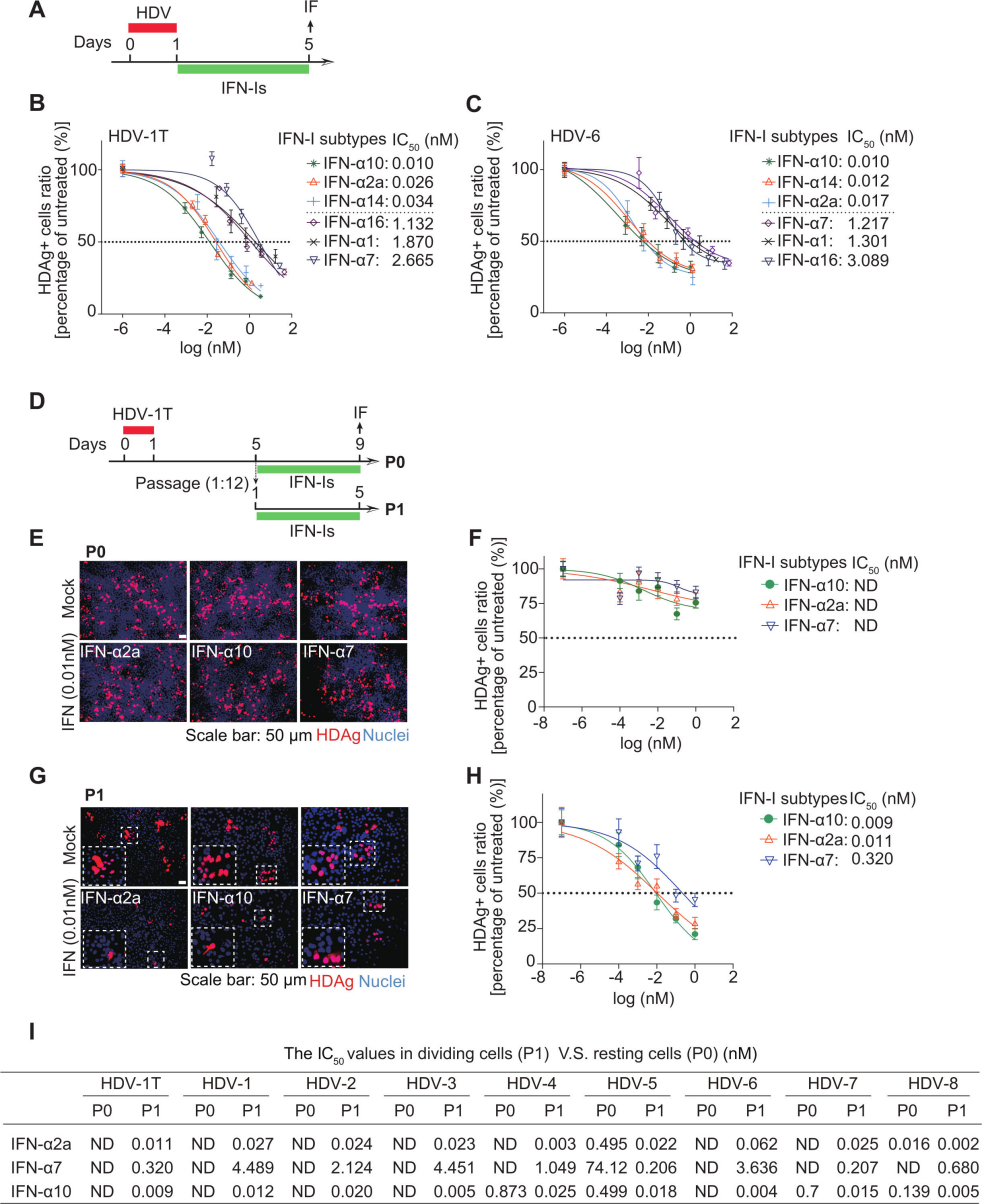
Characterization of the anti-HDV activities of IFN-α subtypes during *de novo* infection, cell mitosis, or cells in resting state. (**A**) Schematic of the experimental setting. Fourteen IFN-I subtypes were added to the culture medium at day 1 post-infection. HDAg-positive cells were imaged and quantified at day 5 post-infection. (**B**) The anti-HDV efficacy of different IFN-I subtypes was examined in HDV-1T-infected HuH7^NTCP^ cells (*n* = 4). (**C**) Same as (**B**) for HDV-6. (**D**) Schematic of the experimental setting. (**E and F**) HuH7^NTCP^ cells were infected with HDV-1T and treated with different doses of IFN-α10, IFN-α2a, or IFN-α7 from day 5 to day 9 (P0, resting cells). HDAg-positive cells were imaged and quantified at day 5 post-infection (*n* = 4). (**G and H**) HuH7^NTCP^ cells were infected with HDV-1T and passaged at a 1:12 dilution (P1, dividing cells). Next, cells were treated with different doses of IFN-α10, IFN-α2a, or IFN-α7 for 5 days, respectively. HDAg-positive cells were imaged and quantified at day 5 post-infection (*n* = 4). (**I**) The IC_50_ values of IFN-α2a, IFN-α10, and IFN-α7 against HDV genotypes in dividing (P1) or resting (P0) cells. ND, not detected.

Humans express 12 IFN-α subtypes with distinct antiviral capabilities against different viruses (31, 32, 38, 49). Currently, only IFN-α2a is widely used to treat chronic HDV infection, leaving the anti-HDV potential of other IFN-α subtypes largely unexplored. Therefore, IFN-α subtypes and two type I IFN subtypes (IFN-β and IFN-ω) were cloned and expressed in HEK293 cells. Their anti-HDV potency was comparatively investigated in three conditions: HDV *de novo* infection (Fig. 5A), HDV in resting cells (Fig. 5D, P0), or in dividing cells (Fig. 5D, P1). During HDV *de novo* infection, all tested IFN-I subtypes significantly inhibited HDV replication in a dose-dependent manner (Fig. 5B and C; Fig. S15 and S16). Notably, varied IC_50_ values were observed against HDV-1T (IC_50_: 10, 2a, 14 < 8, 5, β, 6, 17, ω, 4 < 16, 21, 1, 7) (Fig. 5B; Fig. S15C). Similar results were also obtained in HDV-6 (IC_50_: 10, 14, 2a< ω, 8, 5, β, 21, 17, 4 < 7, 1, 6, 16) (Fig. 5C; Fig. S16C). Of note, IFN-α10, IFN-α2a, and IFN-α14 were the most potent subtypes, whereas IFN-α1, IFN-α16, and IFN-α7 were the least (Fig. 5B and C; Fig. S15C and S16C). Specifically, the IC_50_ values of IFN-α10, IFN-α2a, and IFN-α14 were approximately 100-fold lower than those of IFN-α1, IFN-α16, and IFN-α7 (Fig. 5B and C; Fig. S15C and S16C). Consistently, similar genotype-specific responsiveness was observed in PHH cells (Fig. S17). Subsequently, IFN-α10, IFN-α2a, and IFN-α7 were selected to further evaluate their anti-HDV effects in resting and dividing cells (Fig. 5D). In line with the results of HDV *de novo* infection (Fig. 5B and C), IFN-α10 and IFN-α2a displayed a much stronger anti-HDV effect in dividing cells (P1) compared to IFN-α7 (Fig. 5G and H). Nevertheless, in resting cells (P0), IFN-α10, IFN-α2a, and IFN-α7 were all ineffective against HDV-1T (Fig. 5E and F). Similar results were observed across HDV 1–8 (Fig. 5I; Fig. S18), with the IC_50_ values of IFN-α10 (P1) approximately 13.8 (HDV-7) to 890-fold (HDV-3) lower than those of IFN-α7 (Fig. 5I). In terms of mechanism, IFN-α10 and IFN-α2a triggered higher levels of STAT1 phosphorylation (Fig. S19A) and ISRE activation (Fig. S19B) compared to IFN-α7. Consequently, IFN-α10 and IFN-α2a were significantly more potent than IFN-α7 in the induction of the representative ISGs (Fig. S19C through X). These findings showed that enhanced IFN response by IFN-α10 and IFN-α2a correlated with their superior inhibition of HDV replication, consistent with the central role of JAK/STAT signaling in mediating IFN-I antiviral activity (46, 60). This association supported a functional link between IFN-triggered JAK/STAT activation and anti-HDV efficacy.

### IFN-α subtypes synergize with bulevirtide against HDV

**Fig 6:**
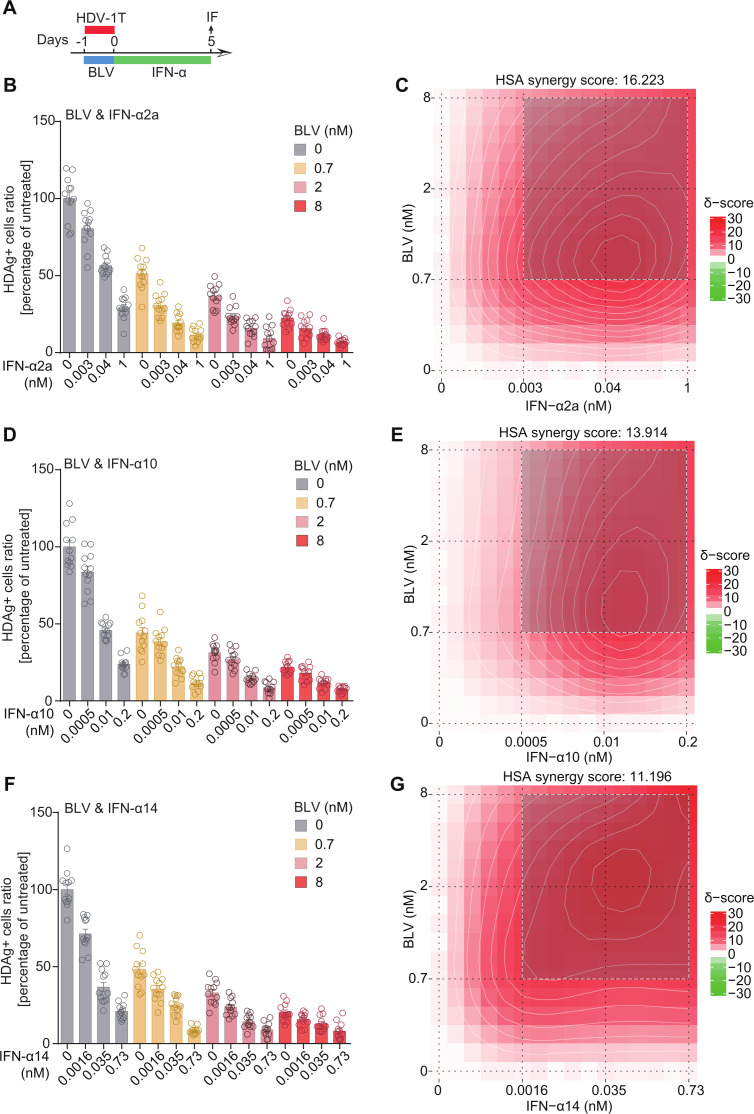
Determination of the synergistic effect in combination between IFN-α subtypes and BLV. (**A**) Schematic of the experimental setting. (**B, D, F**) The anti-HDV efficacy of monotherapy or combination therapies using IFN-α2a (**B**), IFN-α10 (**D**), or IFN-α14 (**F**) with BLV (*n* = 6). (**C, E, G**) Synergy distributions of pairwise combinations of BLV with IFN-α2a (**C**), IFN-α10 (**E**), or IFN-α14 (**G**). HSA, highest single agent. HSA synergy score was calculated by SynergyFinder 3.0 (https://synergyfinder.fimm.fi/). An HSA synergy score >10 indicates a synergistic interaction. The shadow area represents the most synergistic dose window in a dose-response matrix.

In clinical practice, combination therapies were widely employed to enhance antiviral efficacy and mitigate the risk of drug resistance. Previous studies demonstrated that combination therapies using IFN-α2a and BLV exhibited strong synergistic anti-HDV effects, leading to faster and more profound reductions in serum HDV RNA levels during treatment (11, 19–26, 61). Building on this foundation, we further investigated the synergistic potential of additional IFN-α subtypes, specifically IFN-α10 and IFN-α14, in combination with BLV (Fig. 6A). The results revealed that IFN-α10 and IFN-α14, similar to IFN-α2a (Fig. 6B and C), all displayed significant synergy with BLV against HDV (Fig. 6D through G). Notably, most synergistic dose windows were observed at 0.003–1 nM IFN-α2a (Fig. 6C), 0.0005–0.2 nM IFN-α10 (Fig. 6E), and 0.0016–0.73 nM IFN-α14 (Fig. 6G) when combined with 0.7–8 nM BLV, respectively. Consistently, similar results were observed in HuH7^NTCP^ cells infected with HDV-6 (Fig. S20) and HDV-8 (Fig. S21). These results highlight the potential of combining IFN-α10 and IFN-α14 with BLV as a promising therapeutic approach against HDV infection.

## DISCUSSION

Chronic HDV infection results in the most severe form of viral hepatitis. Although HDV was discovered more than 40 years ago, IFN-α2a is currently the only available treatment for HDV infection in most parts of the world (11, 62). Intriguingly, HDV infection is complicated by three different survival strategies: HBV envelope-dependent *de novo* infection, HBV-independent persistence in quiescent cells, and cell division-mediated spread (10, 11). Therefore, it is crucial to thoroughly investigate the effectiveness of IFN-α against HDV in these three different conditions. We found that, unlike the minor effect in resting cells, IFN-α2a showed efficient but varying antiviral potencies against HDV 1-8 isolates during *de novo* infection and cell division. Specifically, upon *de novo* infection, IFN-α2a is most effective against HDV-2 and HDV-8, while the least effective against HDV-6 and HDV-1 (Fig. 1B). In cell-dividing conditions, HDV-8 and HDV-4 are most sensitive to IFN-α2a treatment, whereas HDV-6 and HDV-1 are the least sensitive (Fig. 2H). Interestingly, in resting cells, although all HDV 1–8 isolates were resistant to IFN-α2a, HDV-5 and HDV-8 showed a relatively better response compared to other genotypes (Fig. 2F and H). Thus, the sensitivity of HDV to IFN-α treatment is highly heterogeneous with respect to HDV genotypes and HDV surviving conditions. Notably, the HDV-8 isolate is much more sensitive to IFN-α2a compared to other genotypes in all three different conditions. This *in vitro* finding implies that patients infected with HDV-8 may respond better to IFN-α treatment, but more clinical studies are needed to confirm this observation. Nevertheless, HDV-5 is much more susceptible to IFN-α2a compared to HDV-1 in all three different HDV surviving conditions (Fig. 1B and 2H). Our *in vitro* observation aligns with the finding of a retrospective study in London, which suggests that patients infected with HDV-5 appeared to have a better prognosis and better response to peg-IFN-α2a treatment than patients infected with HDV-1 (31). Similarly, HDV-3 showed a relatively better response to IFN-α2a in comparison to HDV-1, which correlated with a study in Brazil reporting an unusually high response rate (>95%) to peg-IFN-entecavir combination treatment in non-European HDV-3-infected patients (32). We look forward to more clinical retrospective studies to further validate the treatment responsiveness and prognoses of patients with different HDV genotypes following IFN-α2a treatment.

We found that HDV persistence in resting cells was refractory to treatment with IFN-containing cytokine cocktails (Fig. 3D through F). Currently, the underlying mechanisms remain unknown. By contrast, the combinations of IFN-α with extracellular spread inhibitors (e.g., bulevirtide and lonafarnib) could efficiently block both HDV *de novo* infection and cell division-mediated spread (11, 19–28). However, there are currently no available antivirals that efficiently inhibit or even eliminate HDV in resting cells. Understanding the underlying mechanism of HDV resistance in quiescent cells and identifying potent antivirals represents the crucial final stage in eradicating HDV persistence and mitigating post-treatment relapses.

HDV replication is closely related to the editing activity of ADAR1, as it determines the ratios of L-HDAg/S-HDAg (57, 59). Both forms of ADAR1 (p110 and p150) edited the amber/W site to promote the production of L-HDAg, which in turn inhibited HDV replication during *de novo* infection (Fig. S10). Therefore, it is important to thoroughly investigate the connections between IFN-α treatment and ADAR1 protein levels in three different surviving conditions of HDV. We found that HDV in quiescent cells remained resistant to IFN-α treatment, regardless of the levels of p110 and p150 (Fig. 4K through M). Nonetheless, IFN-inducible p150, rather than p110, slightly but significantly contributed to the anti-HDV effect of IFN-α during *de novo* infection and cell mitosis, although the levels of p150 remained lower than the levels of constitutively expressed p110 in the p150 overexpression cells (Fig. S10K). Moreover, IFN-α2a further increased the protein levels of p150 in both OE-ADAR1 p110 and OE-ADAR1 p150 cells, but had no effect on the levels of p110 (Fig. S12). These data highlighted that ADAR1 p150 is one of the important antiviral ISGs (effectors) that contribute to IFN-α2a’s anti-HDV effect.

Among 12 human IFN-α subtypes, IFN-α2a has been extensively studied as a treatment for various viral infections, including chronic HDV infection. In this study, to minimize technical variability, all IFN-α subtypes were produced using a standardized expression and purification protocol, with the same production batch consistently applied across all assays (37, 47, 63). This unified workflow ensures that the observed differences in antiviral activity reflect the intrinsic biological properties of each IFN subtype rather than experimental artifacts. Based on this platform, we found that IFN-α10, IFN-2a, and IFN-14 were the most potent subtypes against HDV, whereas IFN-α1, IFN-16, and IFN-7 were the least during *de novo* infection. We further selected three representative subtypes (IFN-α10, IFN-2a, and IFN-7) from each group and tested their anti-HDV effects in both actively dividing cells and resting cells. Consistent with *de novo* infection, IFN-α10 and IFN-α2a were significantly more potent than IFN-α7 in inhibiting HDV in actively dividing cells, which correlated with their ability to induce ISGs. Therefore, the varied ability in ISG induction is a key factor in determining the effectiveness of different IFN-α subtypes against HDV. In addition, different affinities and/or interaction interfaces within the IFNAR receptor, as well as the difference in the number and spectrum of IFN subtype-specific regulated ISGs all play a role in determining the antiviral potencies of IFN-α subtypes against different viruses (36, 38, 64, 65). Moreover, in clinical practice, combining multiple drugs is a common strategy to enhance antiviral efficacy and mitigate the risk of drug resistance. Our study verified that the most potent subtypes IFN-α10, IFN-α2a, and IFN-α14 all displayed significant synergy in combination with bulevirtide (Fig. 6; Fig. S20 and S21). These results highlight that among all human IFN-α subtypes, IFN-α2a, which is commonly used in clinical settings, is superior against HDV infection. Furthermore, IFN-α10 and IFN-14 hold great potential for the development of alternative IFN-based therapeutic approaches.

Taken together, we demonstrated that the effectiveness of IFN-α in fighting against HDV depends on multiple factors, including HDV genotypes, the levels of IFN-inducible ADAR1 p150 protein, subtypes of IFN-α, and three different HDV surviving strategies. Our study highlights the necessity to understand the mechanisms of HDV resistance in quiescent cells and to develop potent antivirals that specifically target HDV in these resting cells. These efforts are essential in eradicating HDV persistence and reducing the risk of post-treatment relapses.

## Data Availability

The data of this study are available upon reasonable request.

## SUPPLEMENTAL MATERIAL

Supplemental material jvi.01280-25-s0001.docx Figures S1 to S21; Tables S1 and S2.

ASM does not own the copyrights to Supplemental Material that may be linked to, or accessed through, an article. The authors have granted ASM a non-exclusive, world-wide license to publish the Supplemental Material files. Please contact the corresponding author directly for reuse.
