# Supplementary material for: Multimodal characterization of the responsiveness of eight hepatitis D virus genotype isolates to interferon-alpha treatment
Source: J Virol. 2025 Sep 18;99(10):e01280-25. doi: 10.1128/jvi.01280-25 (PMC12548406; doi:10.1128/jvi.01280-25)
Supplement: Supplemental material — Figures S1 to S21; Tables S1 and S2. [file jvi.01280-25-s0001.docx]

**Multimodal characterization of the responsiveness of eight hepatitis D virus genotype isolates to interferon-alpha treatment**

**Table of contents**

Supplementary figures2

Supplementary tables29

**Supplementary figures**

**
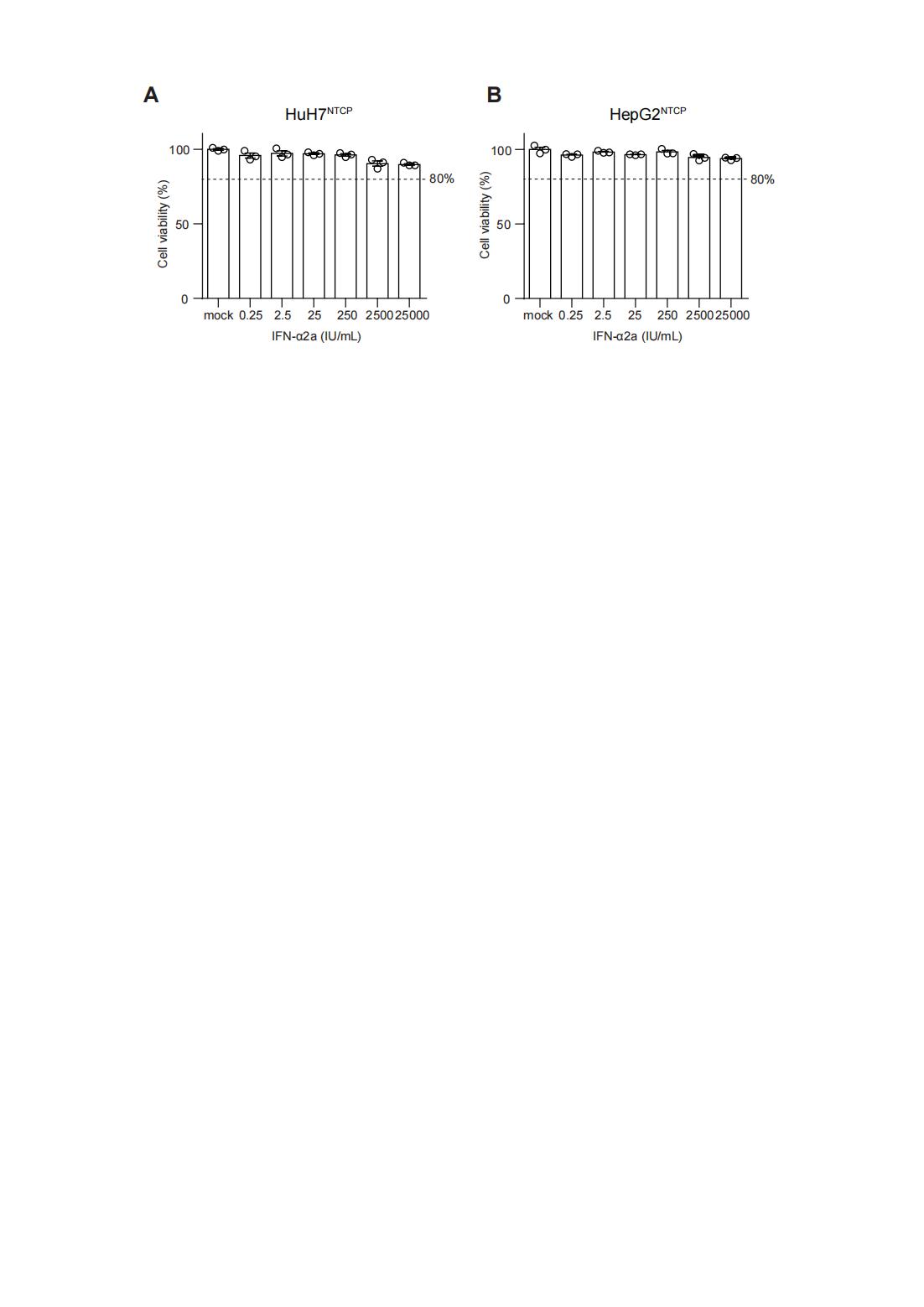
**

**Figure S1** **The WST-1 assay was performed to measure the cytotoxic effects of various concentrations of IFN-α2a on HuH7^NTCP^ and HepG2 ^NTCP^ cells.** HuH7^NTCP^ **(A)** and HepG2^NTCP^ **(B)** cells were incubated with different doses of IFN-α2a (0-25,000 IU/mL) for 48 hours (n=3).


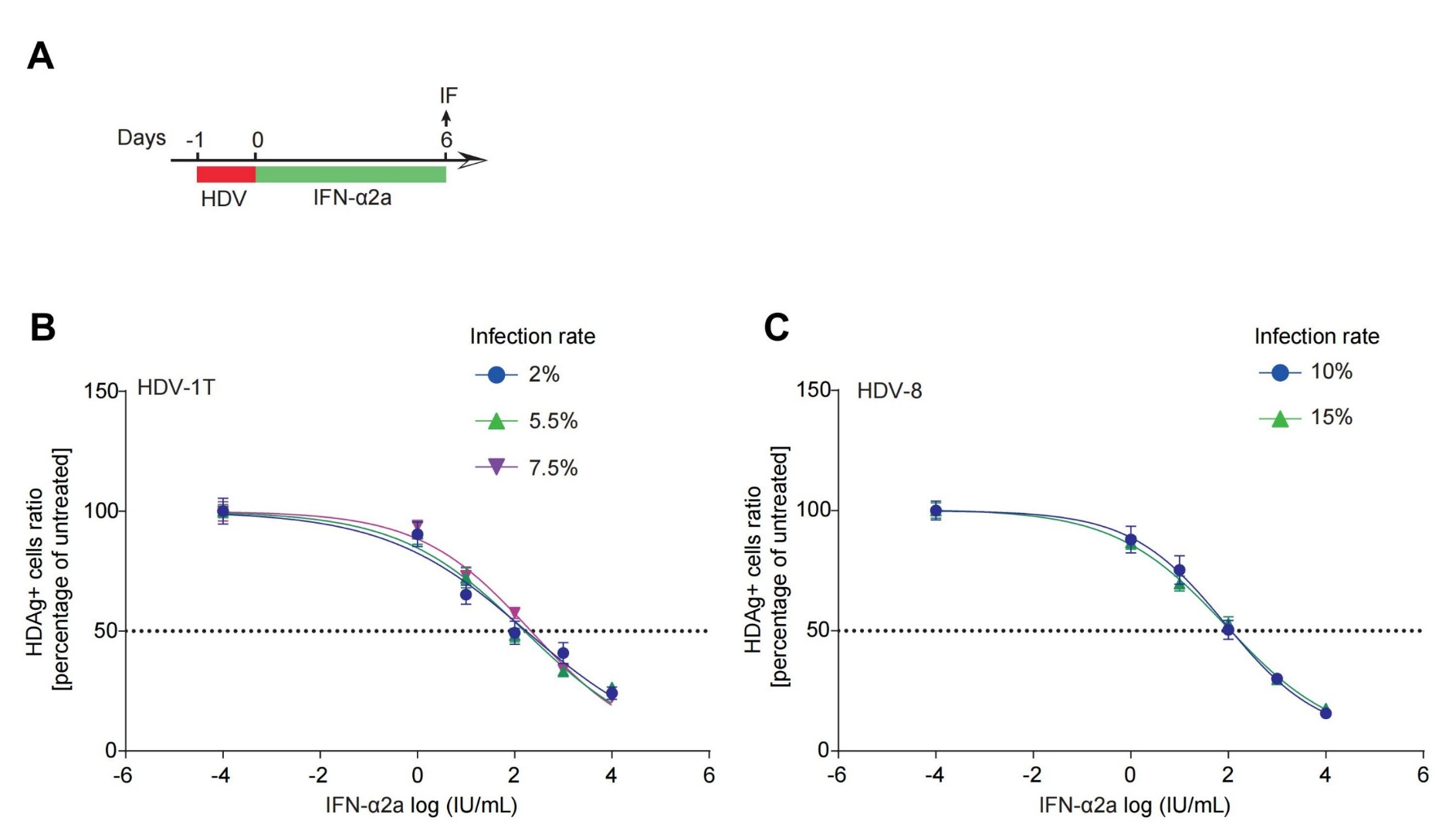


**Figure S2.** **HDV infection rates exert no significant effect on IC_50_ values of IFN-α2a. (A)** Schematic of the experimental setting. **(B, C)** Antiviral activity of IFN-α2a was tested against HDV-1T (B) and HDV-8 (C) in the settings of different HDV infection rate as indicated (n=4).


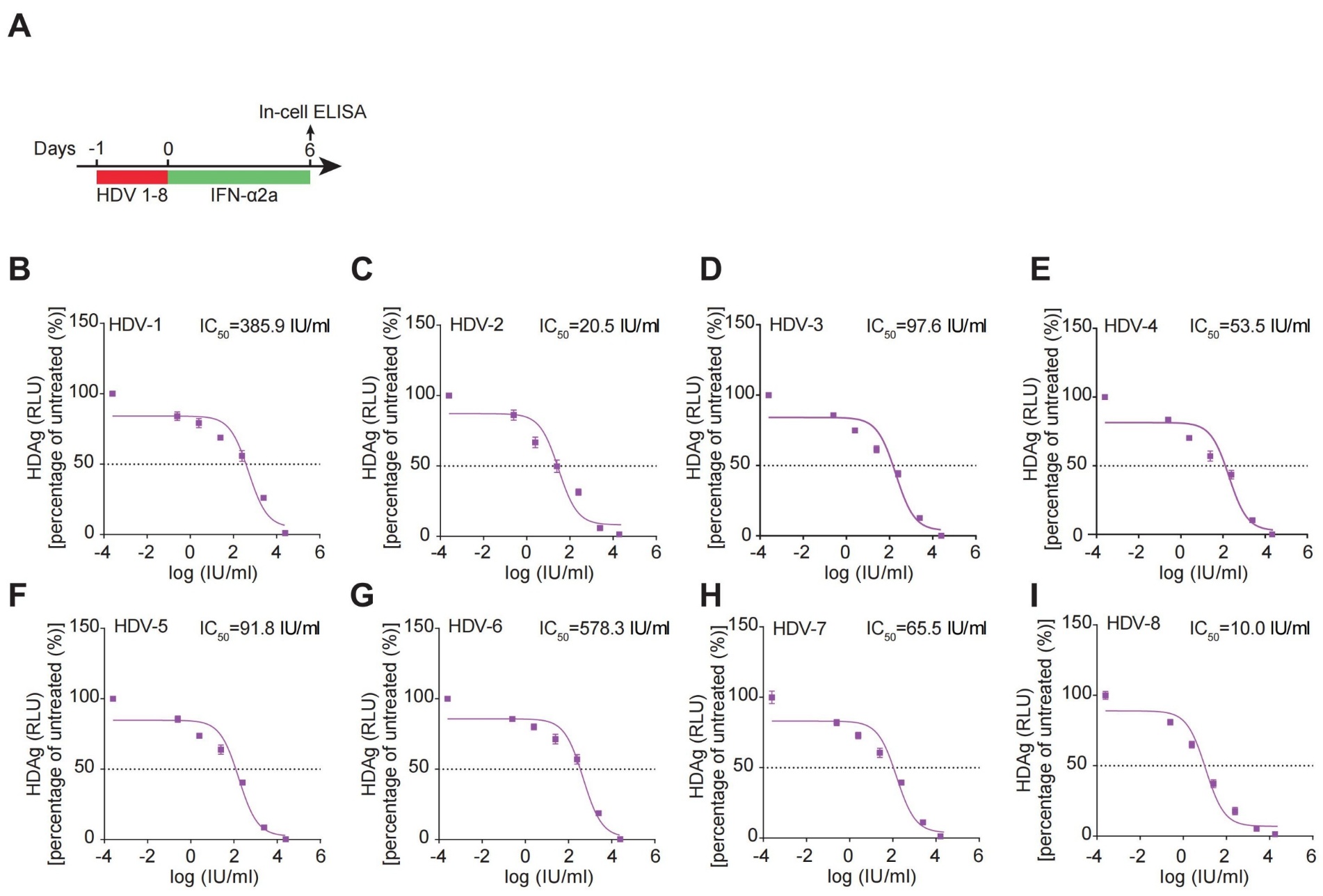


**Figure S3. The anti-HDV efficacy of IFN-α2a in** **HepaRG^NTCP^ cells upon HDV *de novo* infection. (A)** Schematic of the experimental setting. **(B-I)** The antiviral efficacy of IFN-α2a against HDV 1-8 isolates was tested in HepaRG^NTCP^ cells upon HDV *de novo* infection (n=4).


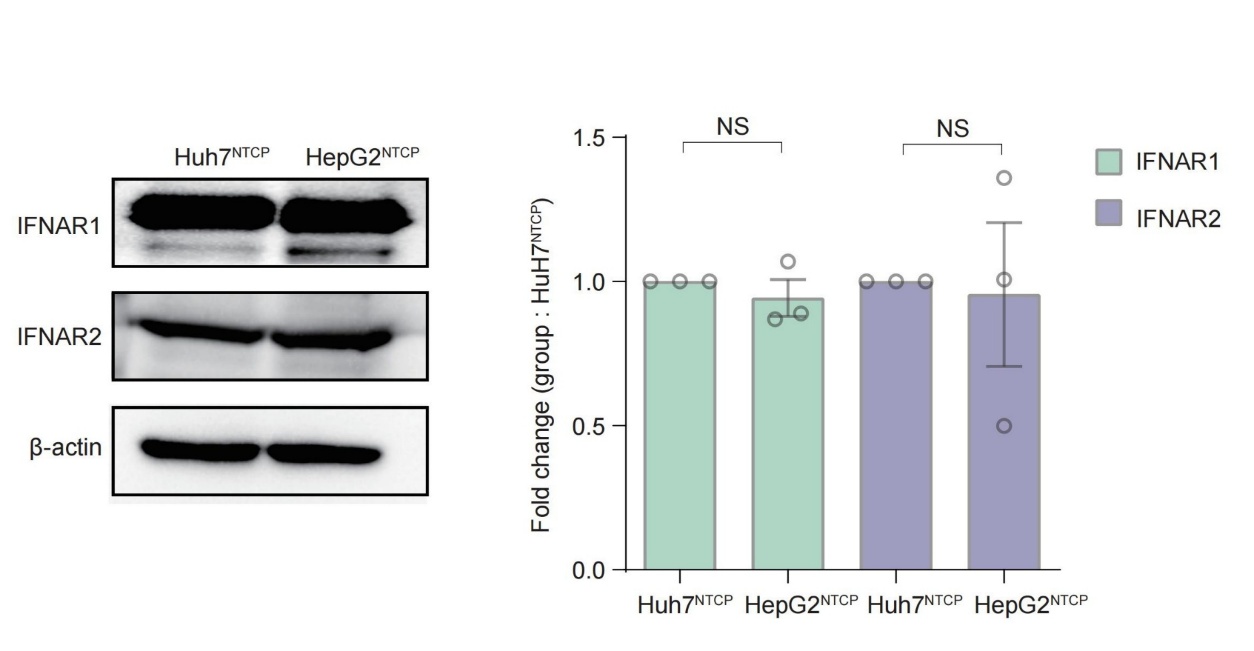


**Figure S4. The levels of IFNAR1 and IFNAR2 in HuH7^NTCP^ and HepG2 ^NTCP^ cells were analyzed by western blot.** HuH7^NTCP^ and HepG2^NTCP^ cells were cultured for 48 hours and the levels of IFNAR1 and IFNAR2 were detected and quantified by western blot (n=3).


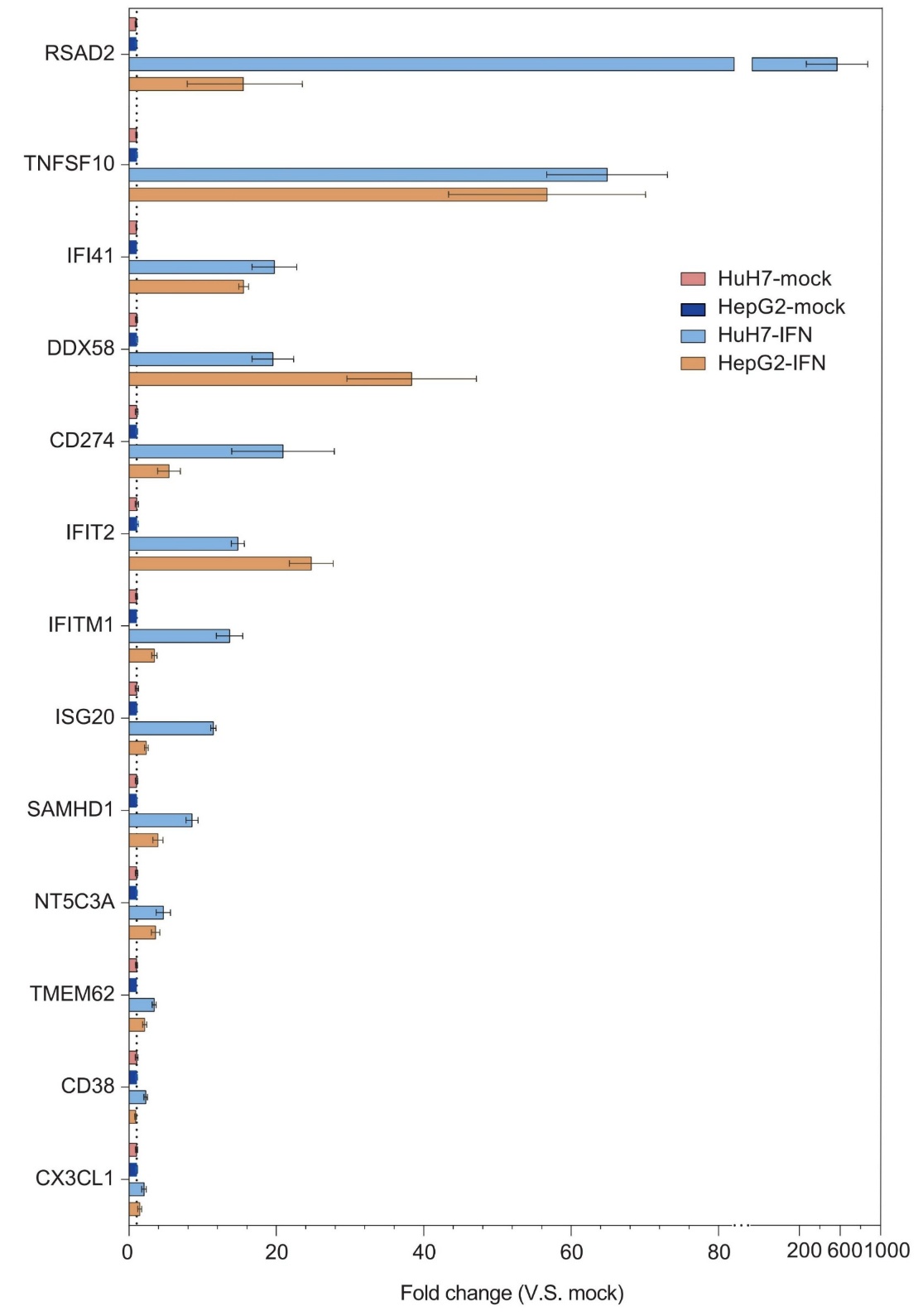


**Figure S5.** **The** **cellular interferon response in HuH7^NTCP^ and HepG2^NTCP^ cells with IFN-α2a treatment.** Huh7^NTCP^ and HepG2^NTCP^ cells were treated with IFN-α2a (1000 IU/mL) for 8 h. A panel of representative ISGs were analyzed by RT-qPCR (n=4).


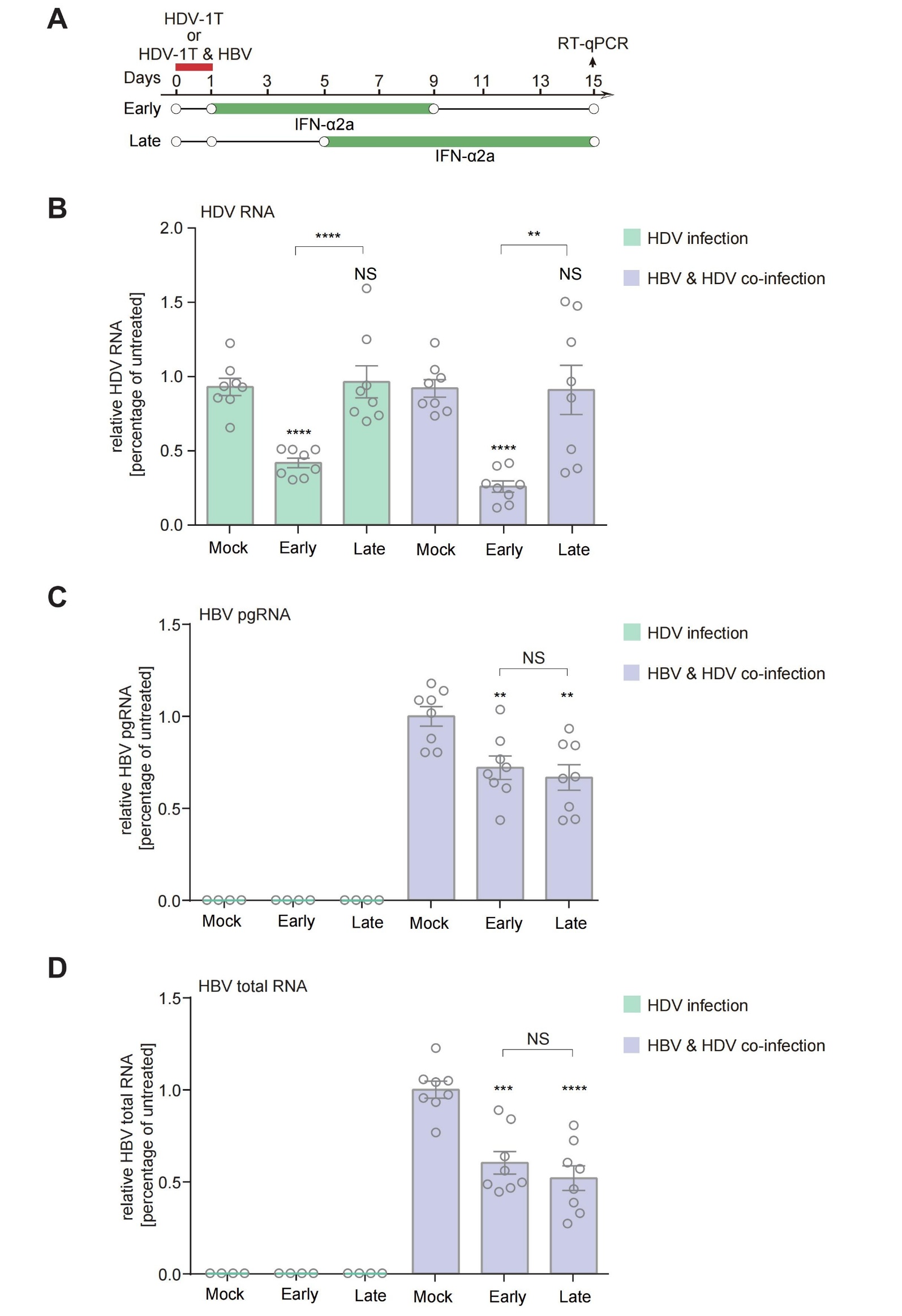


**Figure. S6 Evaluation of the antiviral efficacy of IFN-α2a in HBV-HDV co-infected HuH7^NTCP^ cells upon early or late IFN-α2a treatment. (A)** Schematic of the experimental setting. **(B-D)** The HuH7^NTCP^ cells were infected with HDV-1T alone or co-infected with HDV-1T and HBV. IFN-α2a (1000 IU/mL) was administered at day 1 (early treatment group) or day 5 post-infection (late treatment group). The HDV RNA **(B)**, HBV pgRNA **(C)**, HBV total RNA **(D)** levels were measured by RT-qPCR on day 9 (n=4). ***P* < 0.01, ****P* < 0.001, *****P* < 0.0001, NS: not significant, *P* > 0.05.


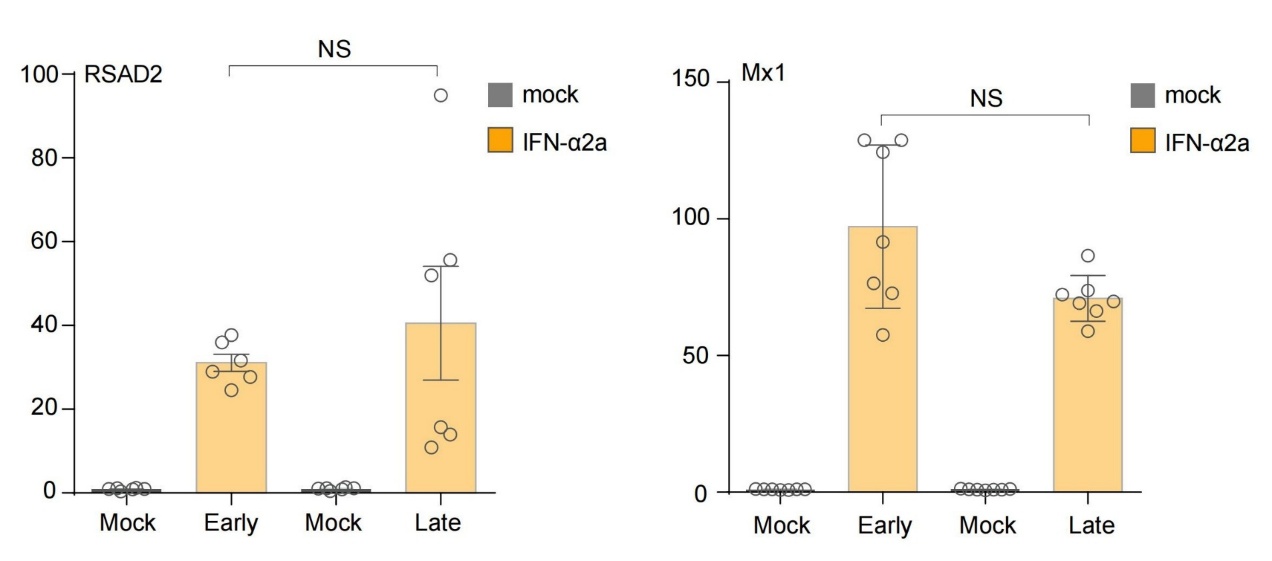


**Figure S7.** **Early or late** **IFN-α2a treatment does not significantly affect the expression levels of ISGs.** HuH7^NTCP^ cells were incubated with IFN-α2a (1000 IU/mL) either in the early treatment group (day 0 post infection) or the late treatment group (day 5 or day 6 post-infection). The mRNA levels of two representative ISGs (RSAD2 and Mx1) were measured by RT-qPCR 48 hours after treatment (n=4). NS: not significant, *P* > 0.05.

**
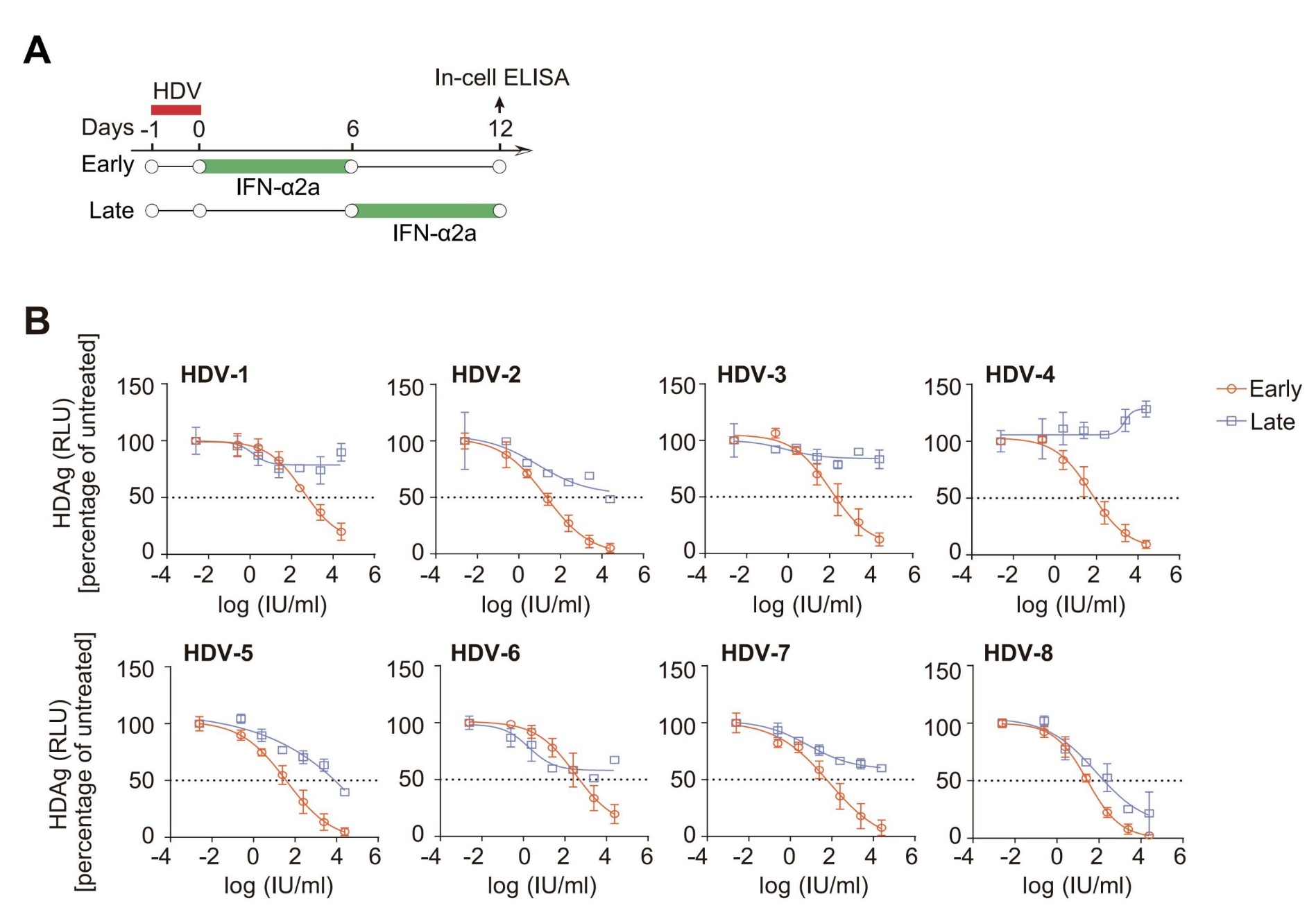
**

**Figure S8 Characterization of the antiviral efficacy of IFN-α2a against HDV 1-8 isolates in resting or dividing cells. (A)** Schematic of the experimental setting. **(B)** HuH7^NTCP^ cells were infected with HDV-1 to HDV-8, respectively. The antiviral efficacy of IFN-α2a against HDV 1-8 were detected by in-cell ELISA in the setting of both early treatment and late treatment (n=4).


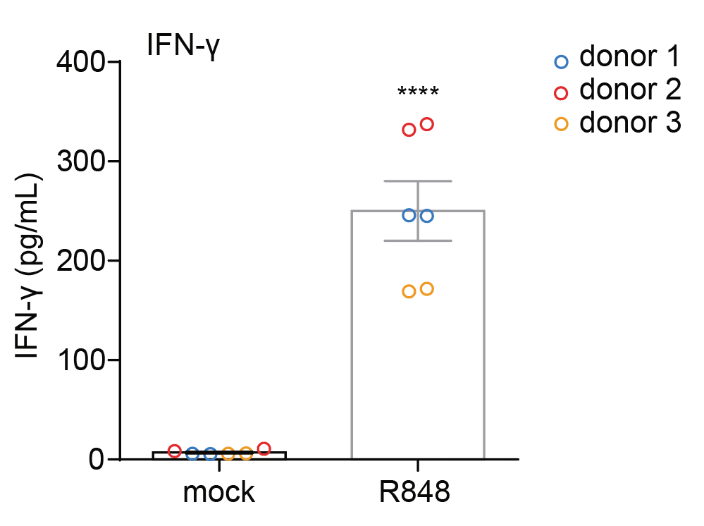


**Figure S9** **The concentration of IFN-γ protein in cytokine cocktail from peripheral blood mononuclear cells (PBMCs) stimulated with R848.** Supernatant concentrations of IFN-γ measured by ELISA at 24 h post R848 stimulation (n = 3). Colors were used to distinguish different participants. *****P* < 0.0001.


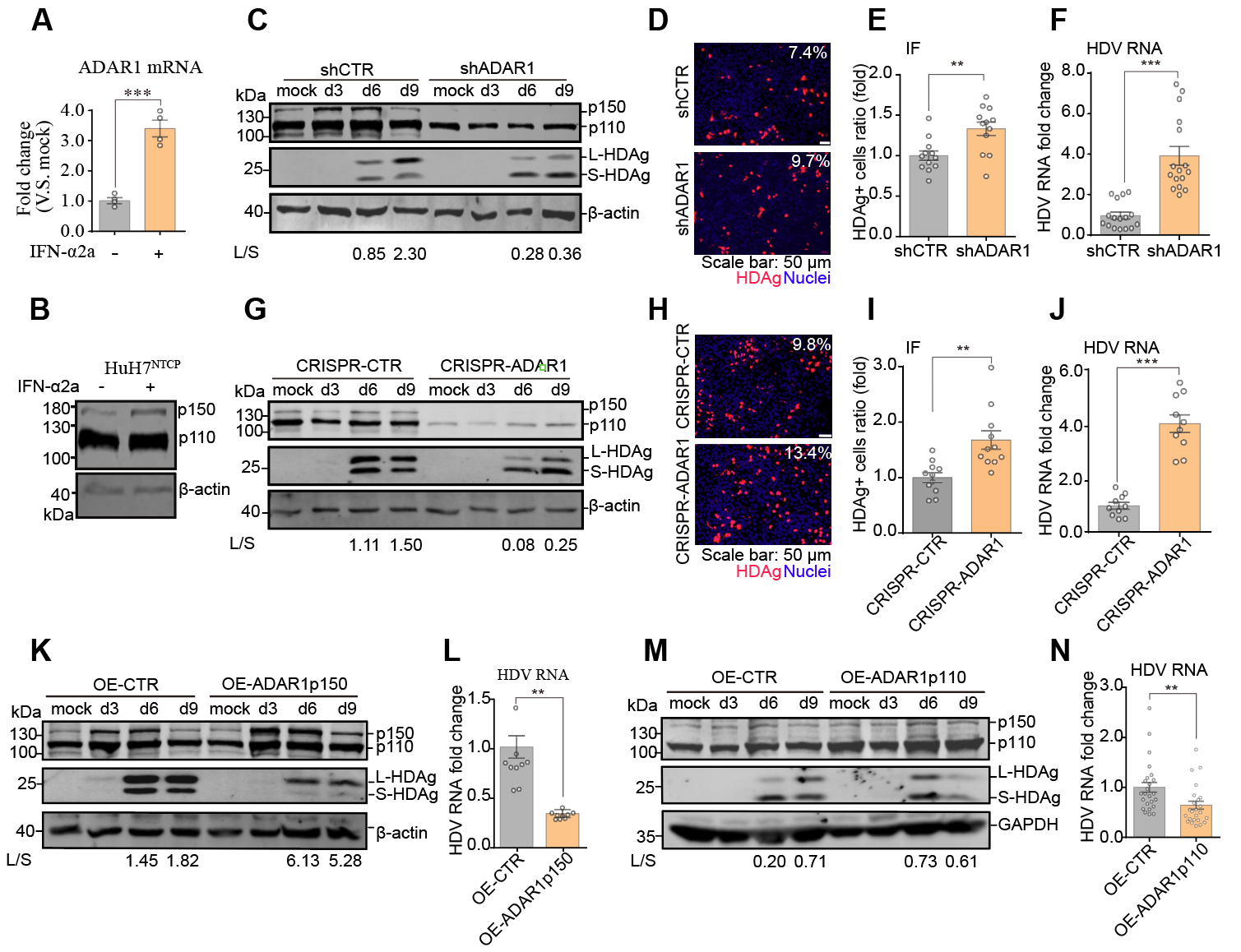


**Figure. S10 The effect of ADAR1 p110 and p150 on HDV replication. (A)** HuH7^NTCP^ cells were treated with IFN-α2a (1000 IU/mL) for 8 h. The mRNA levels of ADAR1 were calculated by RT-qPCR (n=4). **(B)** HuH7^NTCP^ cells were treated with IFN-α2a (1000 IU/mL) for 48 h. The levels of ADAR1 p150 and p110 were detected by western blot. **(C)** shCTR or shADAR1 HuH7^NTCP^ cells were infected with HDV-1T. The levels of ADAR1 p110, ADAR1 p150, L- and S-HDAg were detected by western blot at day 3, 6, and 9 post infection. **(D and E)** The ratios of HDAg positive cells were imaged and quantified at day 9 post infection by IF (n=6). **(F)** The levels of intracellular HDV RNA were quantified by RT-qPCR at day 9, post infection (n=6). **(G)** CRISPR-CTR or CRISPR-ADAR1 HuH7^NTCP^ cells were infected with HDV-1T. The levels of ADAR1 p110, ADAR1 p150, L- and S-HDAg were detected by western blot at day 3, 6, and 9 post infection. **(H and I)** The ratios of HDAg positive cells were imaged and quantified at day 9 post infection by IF (n=6). **(J)** The levels of intracellular HDV RNA were quantified by RT-qPCR at day 9, post infection (n=6). **(K)** The HuH7^NTCP^ overexpression control cells (OE-CTR) and the HuH7^NTCP^ cells with ADAR1 p150 overexpression (OE-ADAR1p150) were infected with HDV-1T. The levels of ADAR1 p110, ADAR1 p150, L- and S-HDAg were assessed by western blot on days 3, 6, and 9 post infection. **(L)** The relative levels of intracellular HDV RNA in OE-CTR and OE-ADAR1p150 cells were quantified by RT-qPCR on day 9 post infection (n=4). **(M)** The OE-CTR and the HuH7^NTCP^ cells with ADAR1 p110 overexpression (OE-ADAR1p110) were infected with HDV-1T. The expression levels of ADAR1 p110, ADAR1 p150, L- and S-HDAg were assessed by western blot on days 3, 6, and 9 post infection. **(N)** The relative levels of intracellular HDV RNA in OE-CTR and OE-ADAR1p110 cells were quantified by RT-qPCR on day 9 post infection (n=12). L/S: L-HDAg/S-HDAg. ***P* < 0.01, ****P* < 0.001.


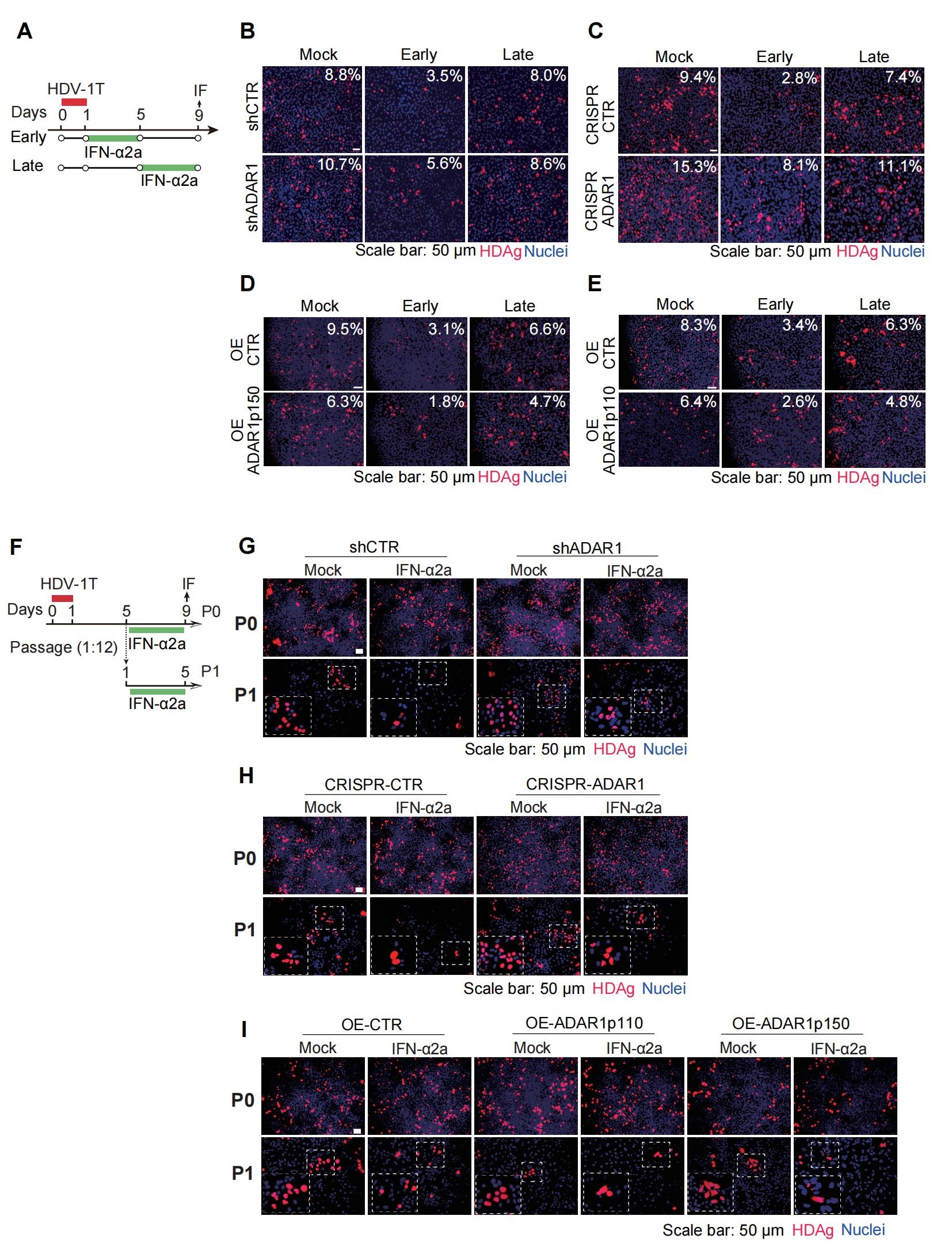


**Figure S11 The roles of ADAR1 p110 and p150 on the anti-HDV efficacy of IFN-α2a.** (A) Schematic of the experimental setting. (B) The shCTR and shADAR1 cells were infected with HDV-1T and then treated with IFN-α2a (1000 IU/ml) from day 1 to day 5 (Early) or from day 5 to day 9 (Late). HDAg-positive cells were imaged and quantified at day 9 post infection (n=6). (C) Same as (B) for CRISPR-CTR and CRISPR-ADAR1 cells. (D) Same as (B) for OE-CTR and OE-ADAR1p150 cell. (E) Same as (B) for OE-CTR and OE-ADAR1p110 cells. (F) Schematic of the experimental setting. (G) The shCTR and shADAR1 cells were infected with HDV-1T. Cells were either passaged at a 1:12 dilution (P1, dividing cells) or left without passaging (P0, resting cells). HDAg positive cells were imaged and quantified 5 days post IFN treatment (1000 IU/ml) by IF (n=4). (H) Same as (G) for CRISPR-CTR and CRISPR-ADAR1 cells. (I) Same as (H) for OE-CTR, OE-ADAR1p150, and OE-ADAR1p110 cells.


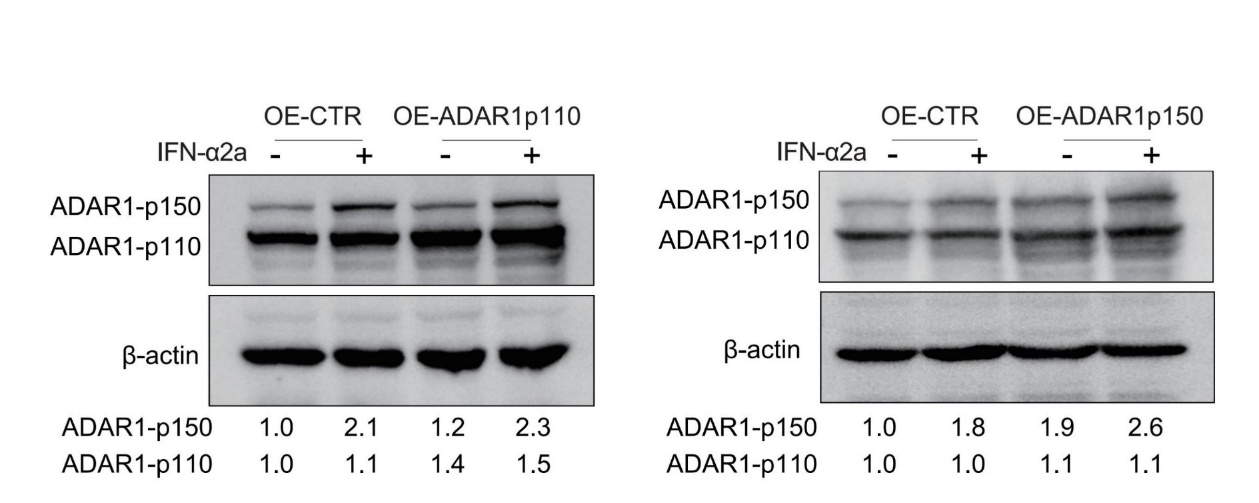


**Figure S12. Determination of the expression level of ADAR1 p110 and p150 of OE-CTR, OE-ADAR1 p110 and OE-ADAR1 p150 cells with IFN-α2a** OE-CTR, OE-ADAR1p110 and OE-ADAR1p150 cells were treated with IFN-α2a (1000 IU/mL) for 48 h. The levels of ADAR1p150 and p110 were detected by western blot.

**
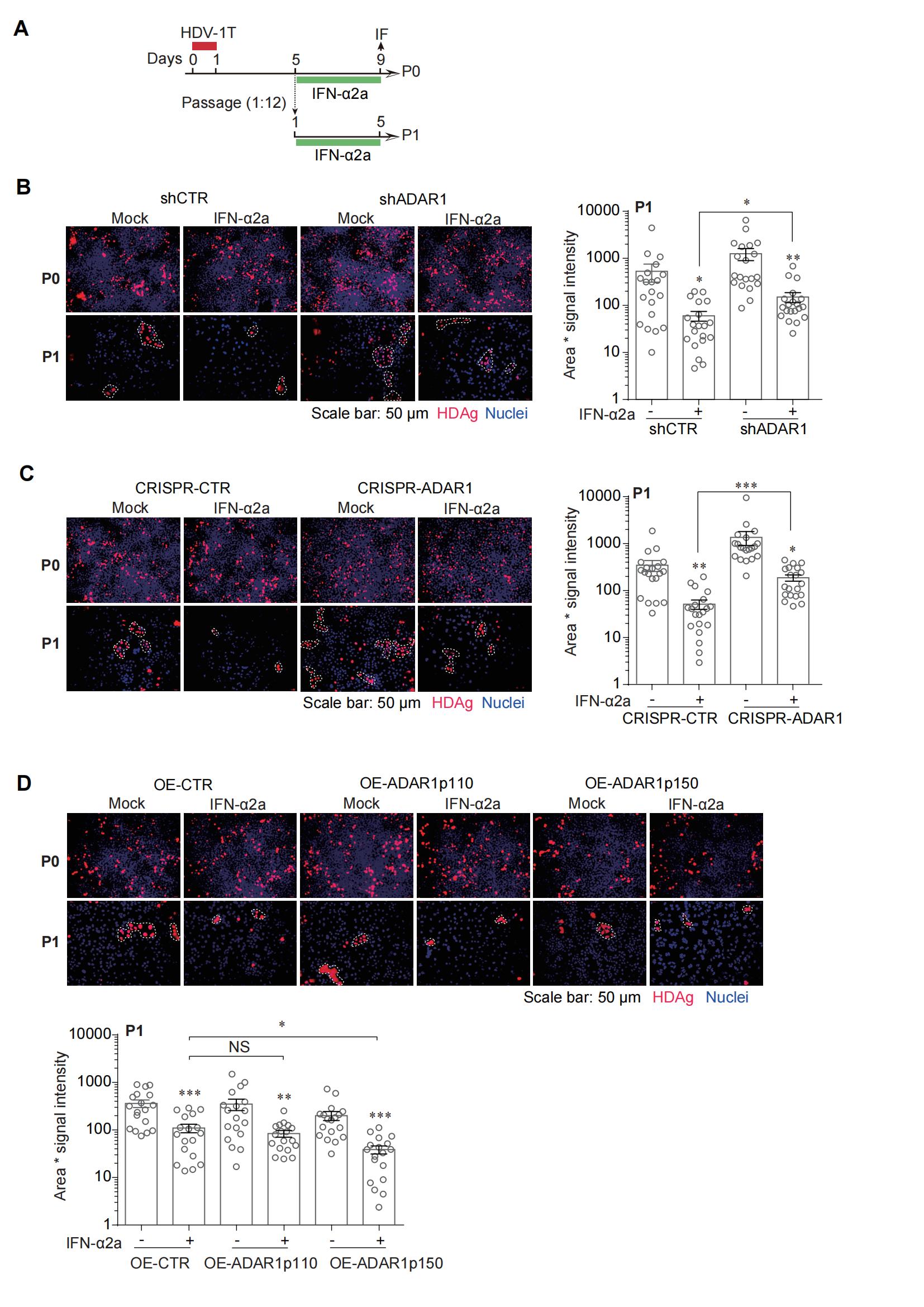
**

**Figure. S13 Characterization of the antiviral efficacy of IFN-α2a against HDV in resting or dividing cells.** Quantitative assessment of HDAg-positive cluster sizes in ADAR1-overexpressing and knockout cell lines following IFN-α2a treatment. The images used for this analysis are identical to those presented in Fig. S11 but were reanalyzed using ImageJ to quantify cluster area and integrated intensity, providing an independent metric of HDV spread via cell mitosis. ***P* < 0.01, ****P* < 0.001, NS: not significant, *P* > 0.05.


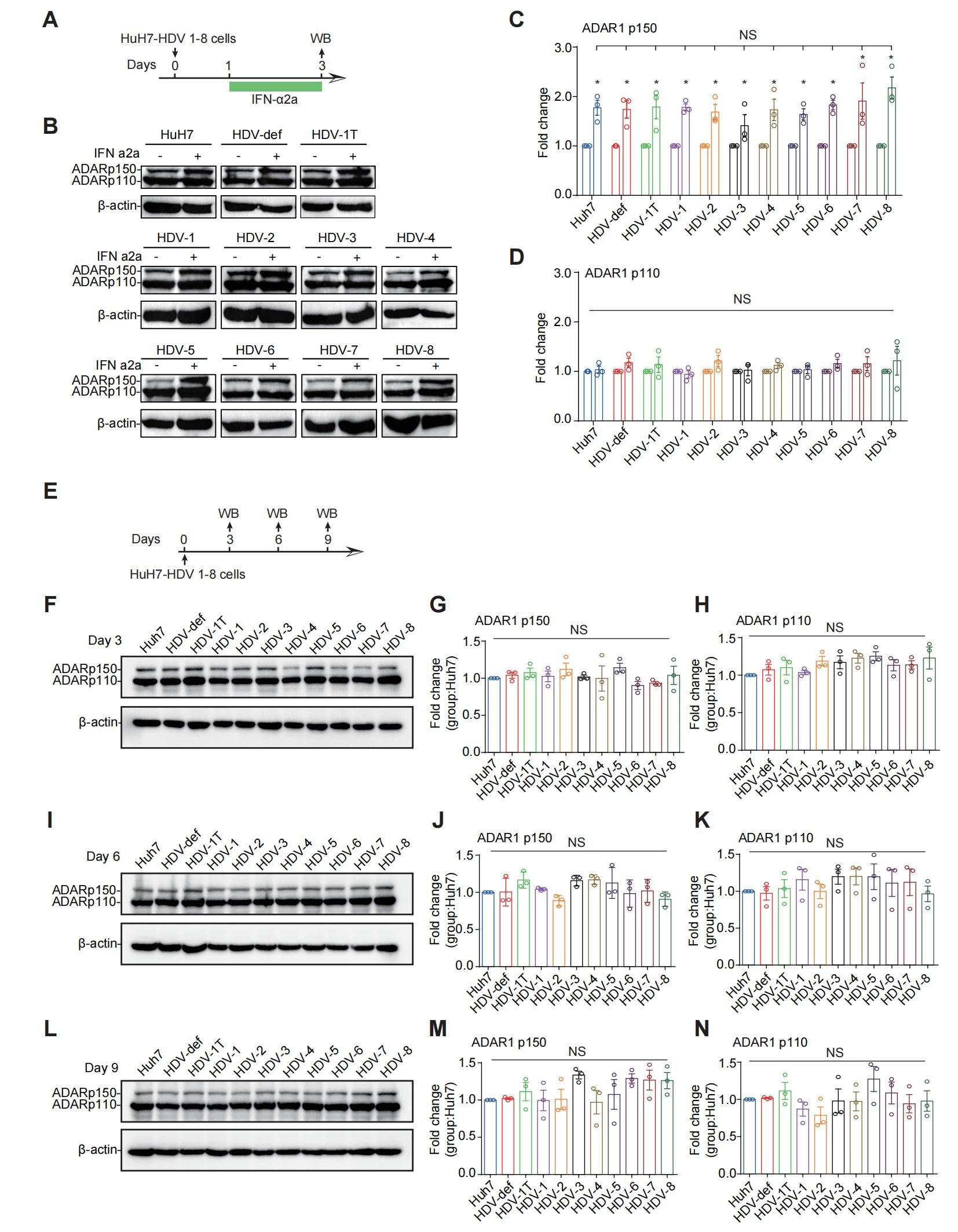


**Figure. S14 Determination of the expression level of ADAR1 p110 and p150 in the presence of HDV 1-8 replication. (A)** Schematic of the experimental setting. **(B-D)** The levels of ADAR1 p150 **(C)** and p110 **(D)** were detected by western blot with IFN-α2a (1000 IU/mL) treatment for 48 h. HDV-def: replication-defective HDV. **(E)** Schematic of the experimental setting. **(F-H)** The expression levels of ADAR1 p150 **(G)** and ADAR1 p110 **(H)** were detected by western blot at day 3 **(F)**. **(I-K)** The expression levels of ADAR1 p150 **(J)** and ADAR1 p110 **(K)** were detected by western blot at day 6 **(I)**. **(L-N)** The expression levels of ADAR1 p150 **(M)** and ADAR1 p110 **(N)** were detected by western blot at day 9 **(L)**. **P* < 0.05, NS: not significant, *P* > 0.05.


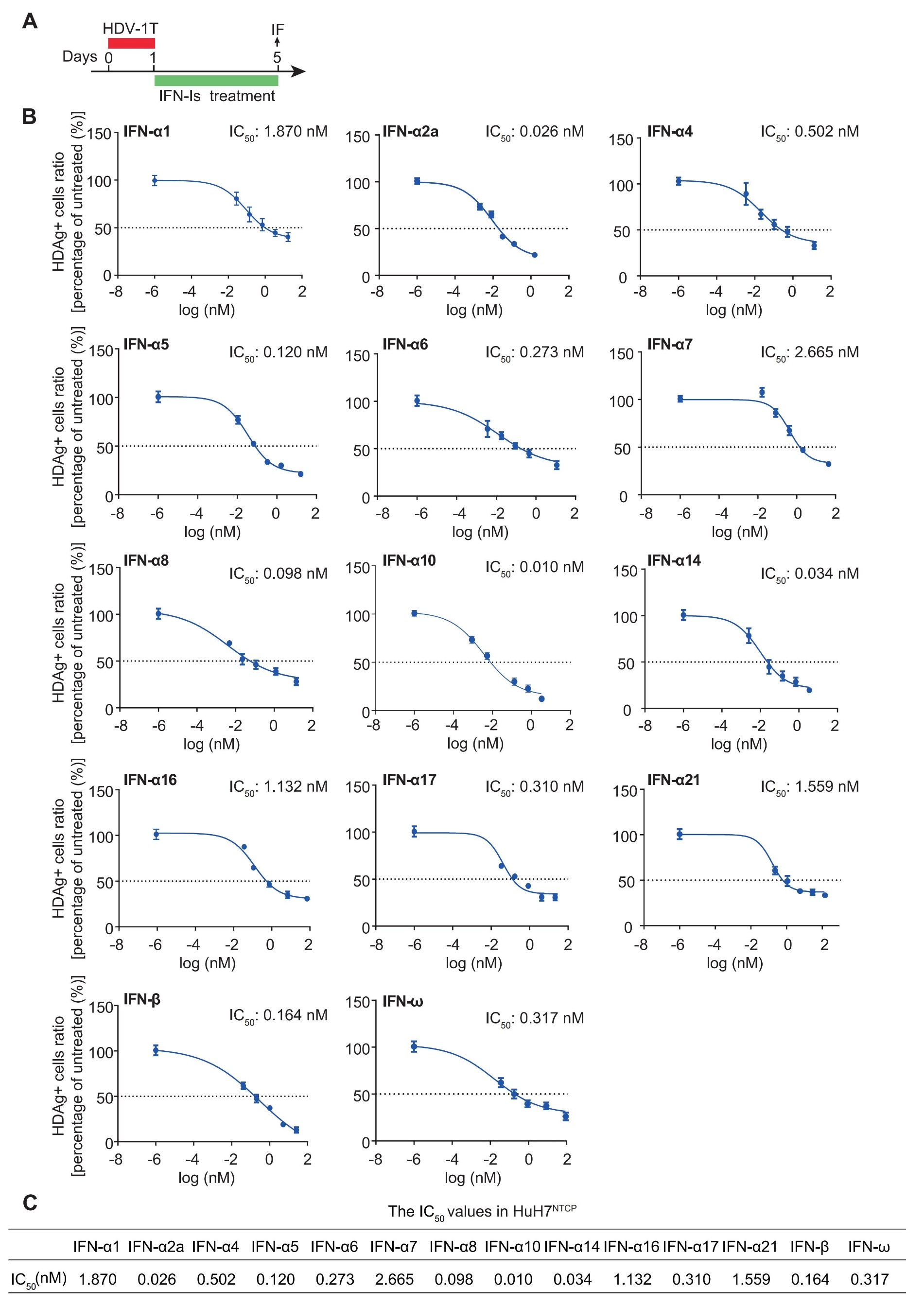


**Figure S15 The antiviral efficacy of 14 IFN-I subtypes in HuH7^NTCP^ cells upon HDV-1T *de novo* infection. (A)** Schematic of the experimental setting. **(B-O)** The anti-HDV efficacy of different IFN-I subtypes were examined in HDV-1T infected HuH7^NTCP^ cells (n=4). **(P)** The IC_50_ values of different IFN-I subtypes against HDV-1T.

**
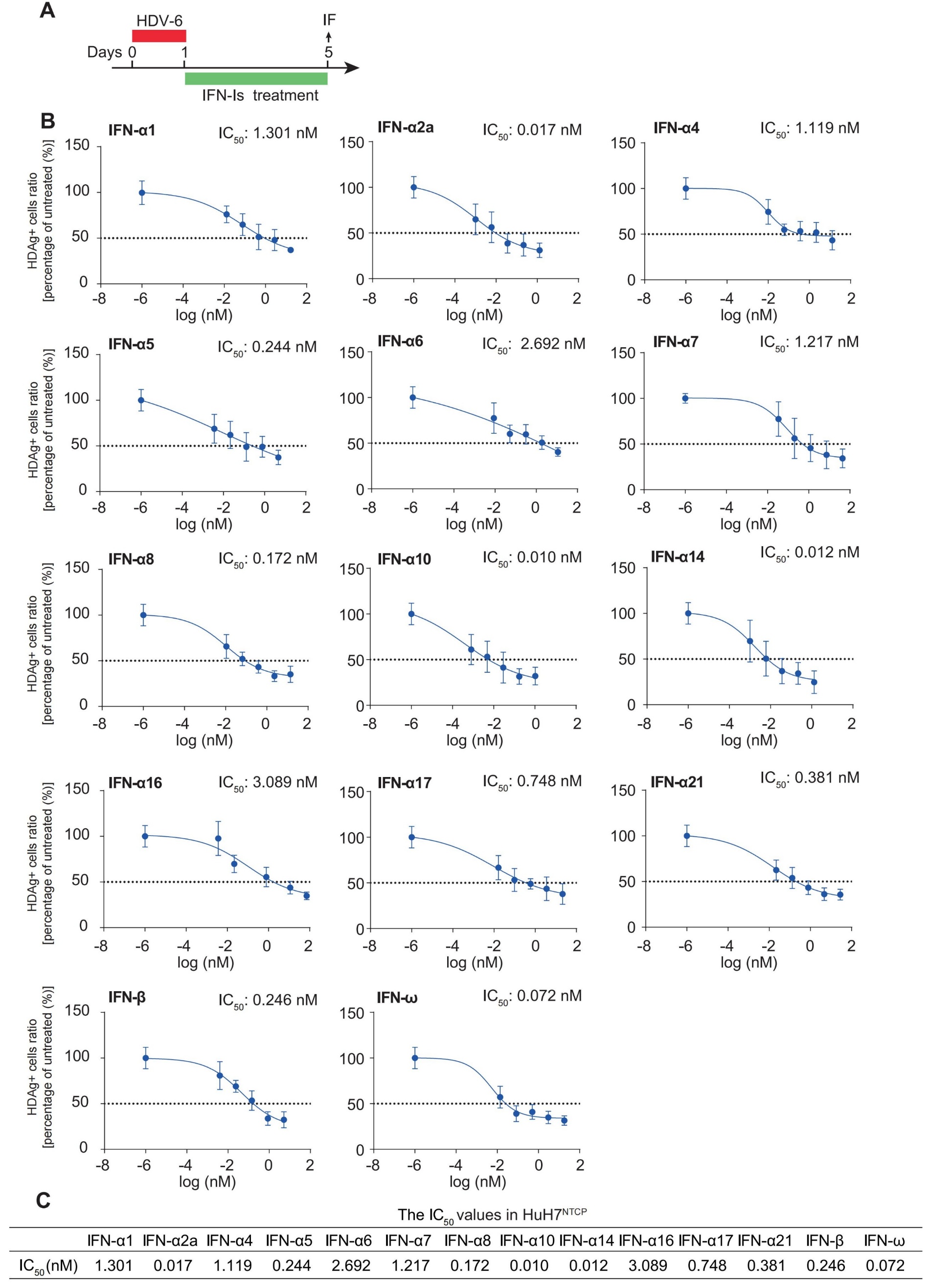
**

**Figure S16 The antiviral efficacy of 14 IFN-I subtypes in HuH7^NTCP^ cells upon HDV-6 *de novo* infection. (A)** Schematic of the experimental setting. **(B-O)** The anti-HDV efficacy of different IFN-I subtypes were examined in HDV-6 infected HuH7^NTCP^ cells (n=4). **(P)** The IC_50_ values of different IFN-I subtypes against HDV-6.


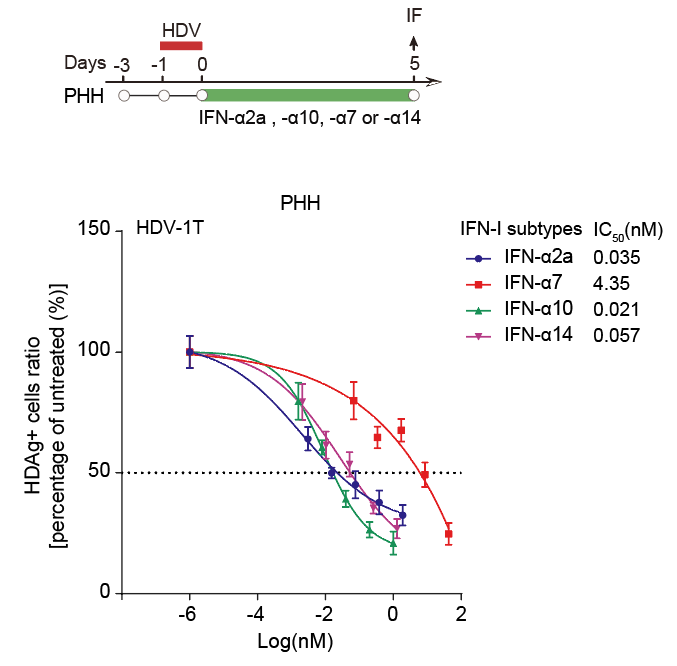


**Figure S17 Characterization the anti-HDV activities of IFN-α subtypes in primary human hepatocytes (PHH).** PHH were seeded at a density of 1.5×10⁵ cells/cm² and infected with HDV-1T. After 16 hours of HDV-1T infection, the cells were washed twice with maintenance medium, and then treated with IFN-α10, -α2a, -α7 or -α14 from day 1 to day 5. HDAg-positive cells were imaged and quantified at day 5 post infection.


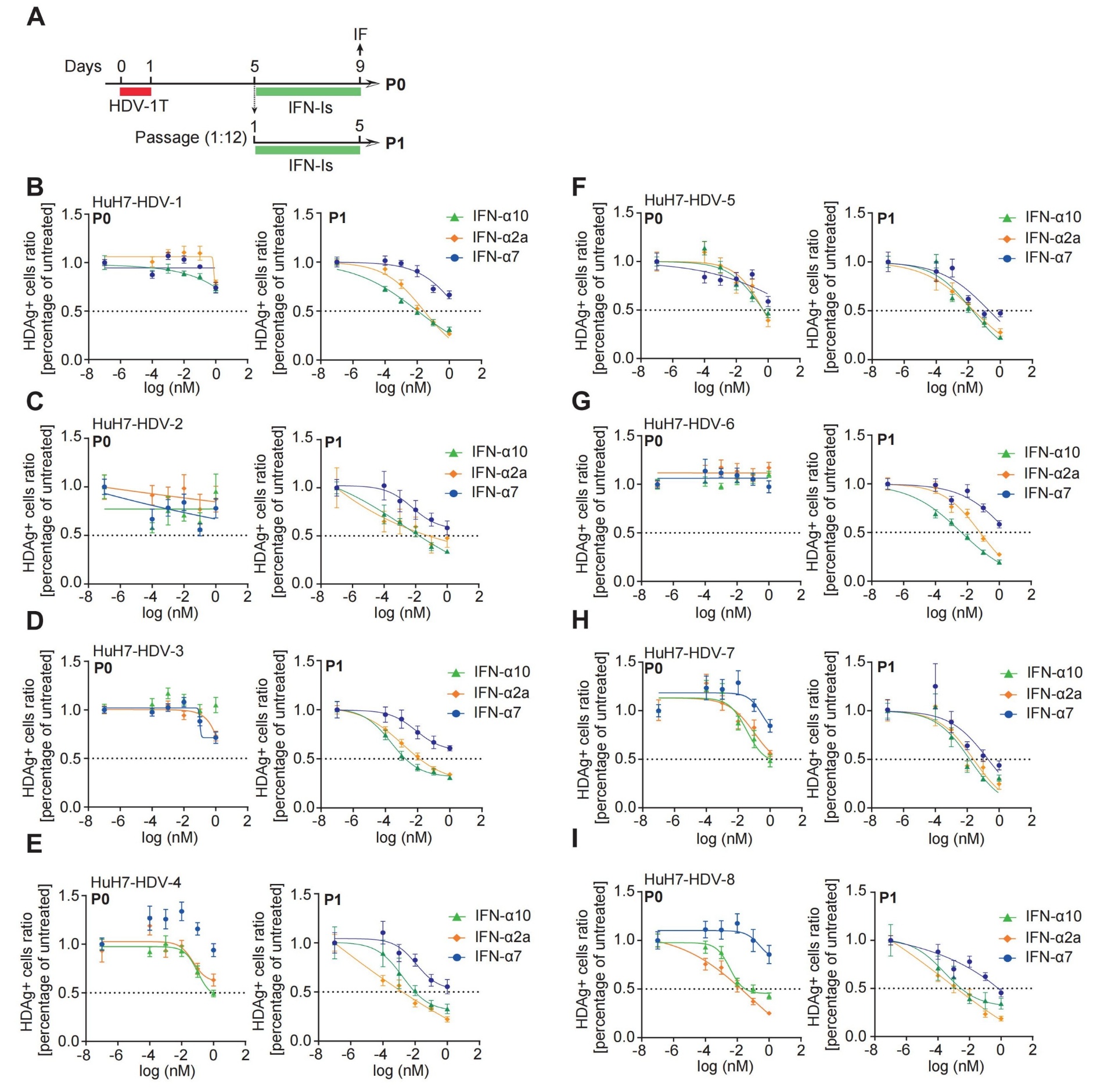


**Figure S18 Characterization the anti-HDV activities of IFN-α subtypes during cell mitosis or cells in resting state. (A)** Schematic of the experimental setting. **(B-I)** HuH7-HDV-1 to 8 cells were either passaged at 1:12 dilution (P1, dividing cells) or left without passaging (P0, resting cells). Next, cells were treated with IFN-α2a, IFN-α10, and IFN-α7 for 5 days. HDAg-positive cells were quantified at day 5 post treatment (n=4).


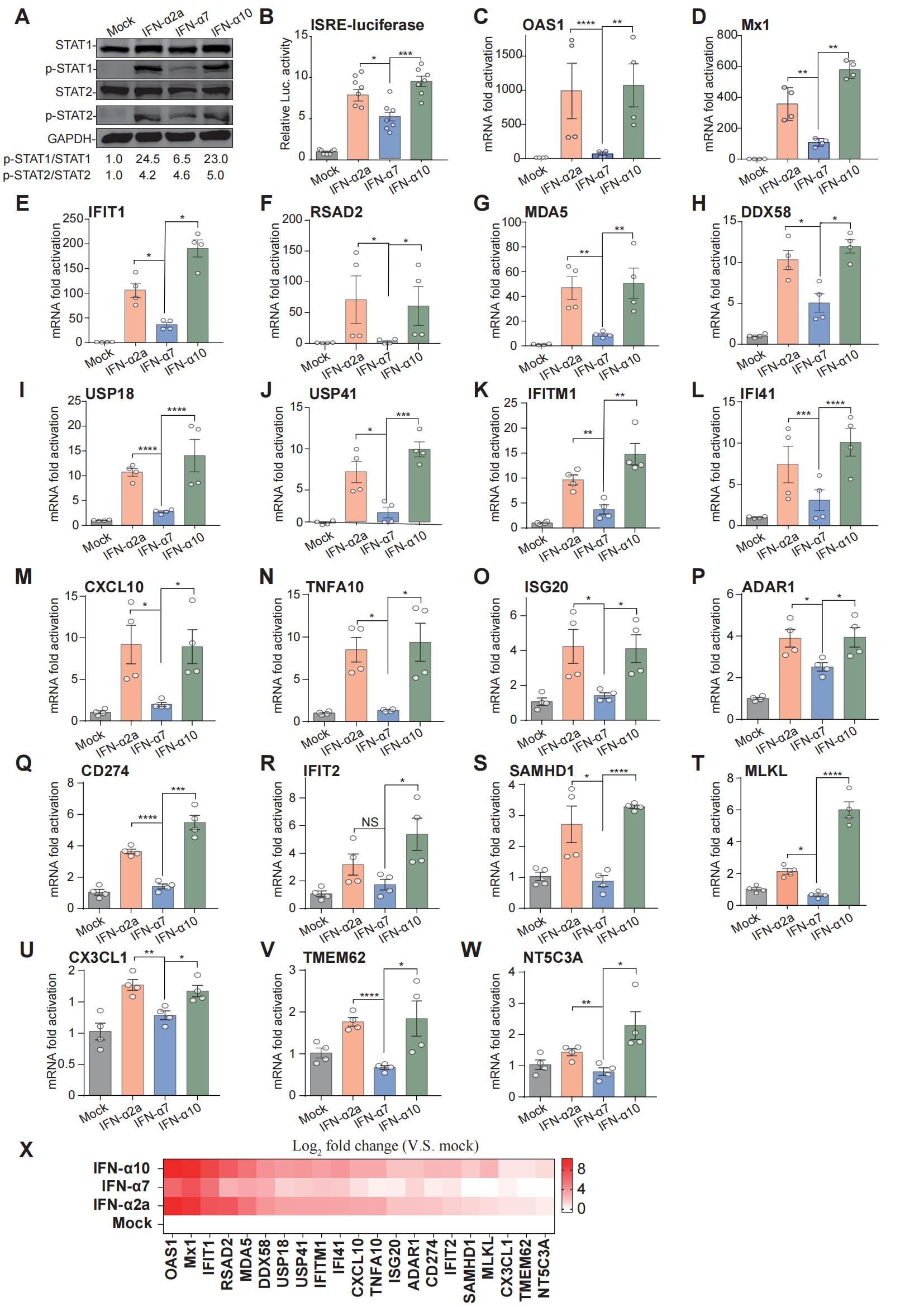


**Figure S19.** **The cellular interferon response of Huh7^NTCP^ cells to IFN-α2a, IFN-α7, or IFN-α10 treatment. (A)** HuH7^NTCP^ cells were treated with IFN-α2a, IFN-α7 and IFN-α10 (0.1 nM) for 30 mins. The levels of STAT1, STAT2, pSTAT1(Y701), pSTAT2(Y690) were detected by western blots. The ratios of p-STAT1/STAT1 or p-STAT2/STAT2 were calculated by normalizing IFN-α2a-induced STAT1 or STAT2 phosphorylation levels to both GAPDH and the baseline STAT1 or STAT2 expression. **(B)** HuH7^NTCP^ cells were treated with IFN-α2a, IFN-α7, and IFN-α10 (0.1 nM) for 8 hours. The relative levels of ISRE luciferase activity were detected (n=7). **(C-X)** HuH7^NTCP^ cells were treated with IFN-α2a, IFN-α7, and IFN-α10 (0.003 nM) for 8 hours. A panel of representative ISGs were analyzed by RT-qPCR, and the data were presented either in the bar chart **(C-W)**, heatmap **(X)** (n=4). ***P* < 0.01, ****P* < 0.001, *****P* < 0.0001.

**
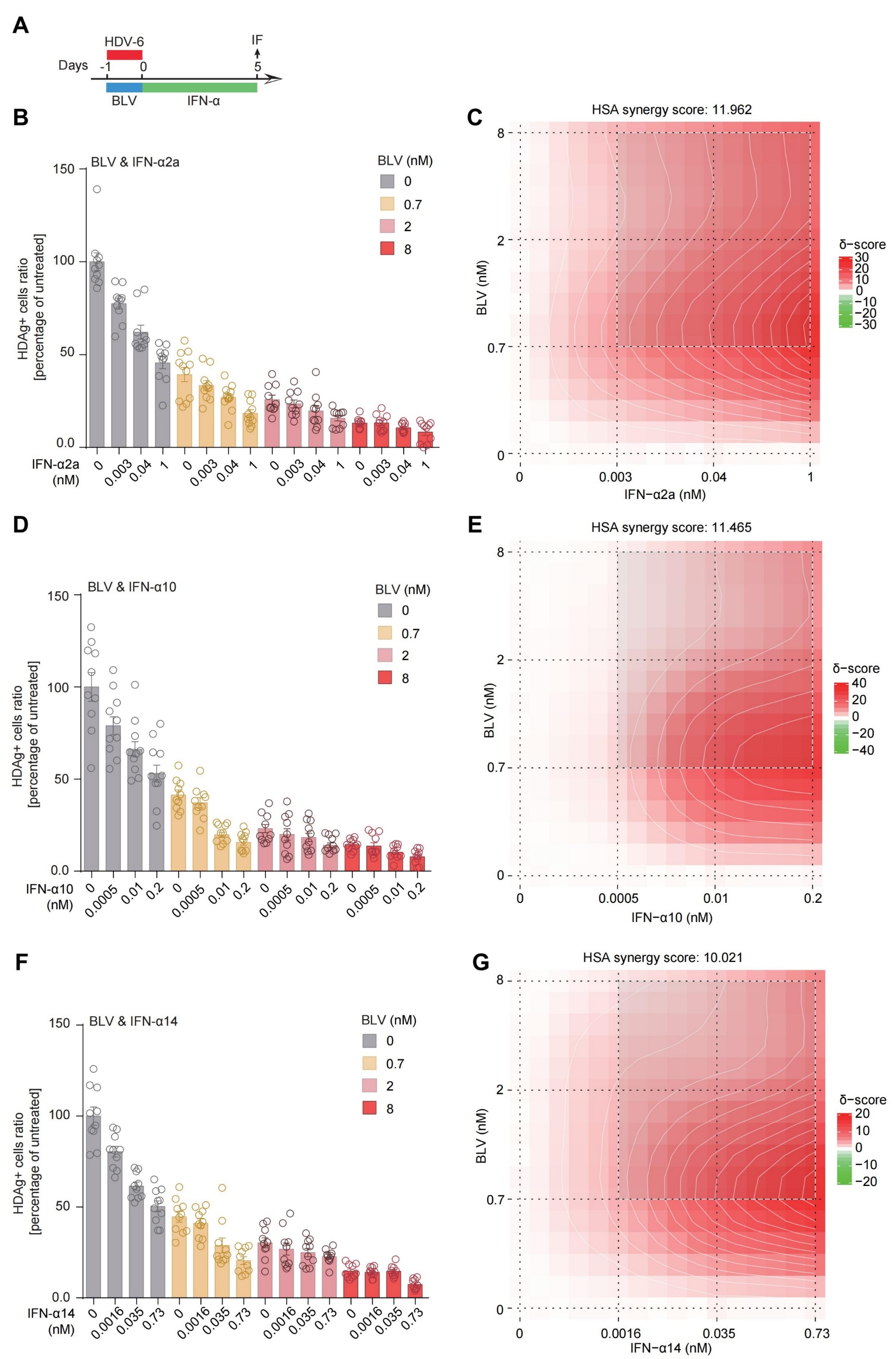
**

**Figure S20. Determination of the synergistic effect in combination between IFN-α subtypes and BLV. (A)** Schematic of the experimental setting. **(B, D, F)** The anti-HDV efficacy of monotherapy or combination therapies using IFN-α2a **(B)**, IFN-α10 **(D)**, or IFN-α14 **(F)** with BLV (n=6). **(C, E, G)** Synergy distributions of pairwise combinations of BLV with IFN-α2a **(C)**, IFN-α10 **(E)**, or IFN-α14 **(G)**. Synergy score was calculated by SynergyFinder 3.0 (https://synergyfinder.fimm.fi/). HSA, highest single agent. An HSA score >10 indicates a synergistic interaction. The shadow area represents the most synergistic dose-window in a dose-response matrix.


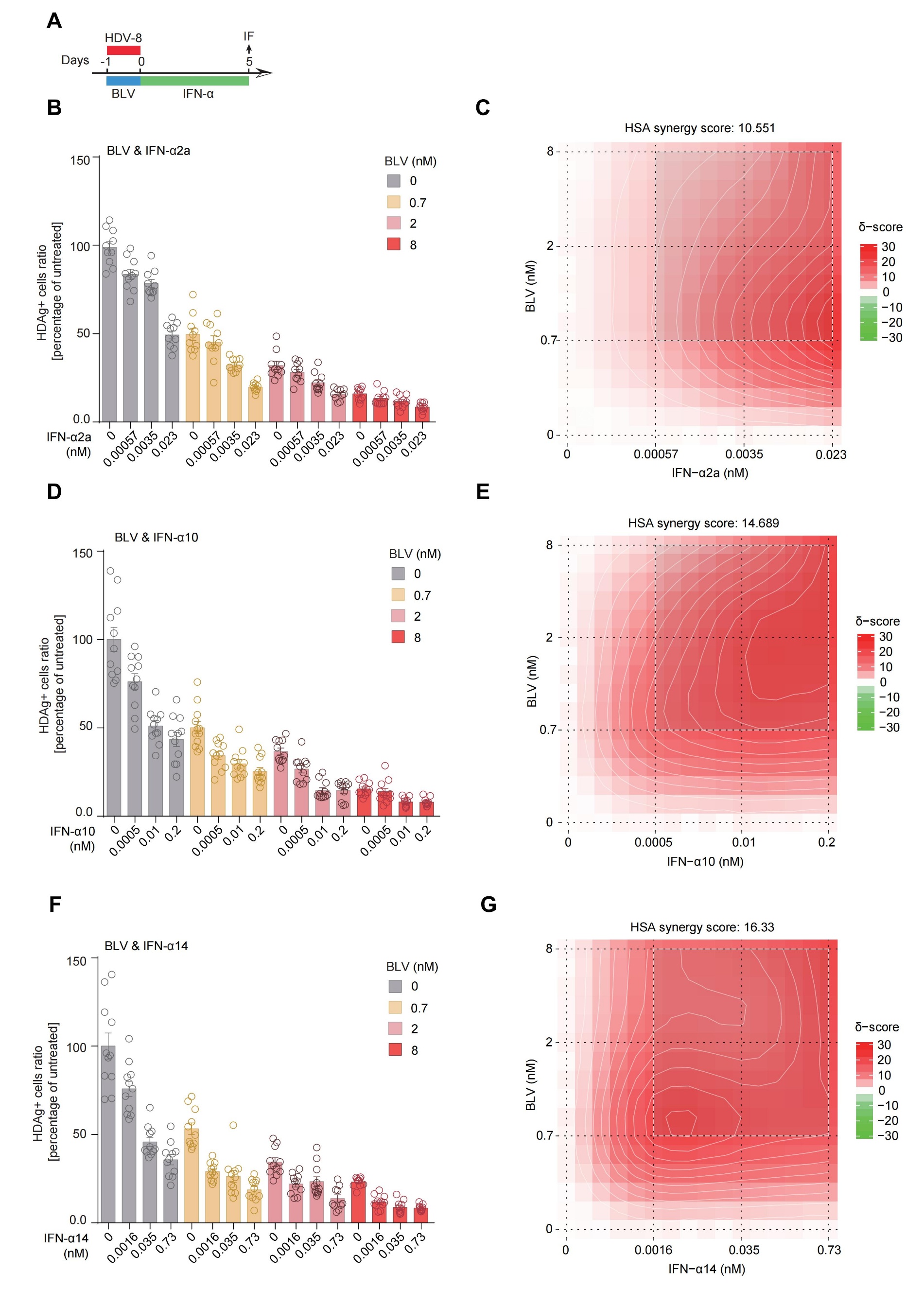


**Figure S21 Determination of the synergistic effect in combination between IFN-α subtypes and BLV**. (A) Schematic of the experimental setting. (B, D, F) The anti-HDV efficacy of monotherapy or combination therapies using IFN-α2a (B), IFN-α10 (D), or IFN-α14 (F) with BLV (n=6). (C, E, G) Synergy distributions of pairwise combinations of BLV with IFN-α2a (C), IFN-α10 (E), or IFN-α14 (G). HSA, highest single agent. HSA synergy score was calculated by SynergyFinder 3.0 (https://synergyfinder.fimm.fi/). An HSA synergy score >10 indicates a synergistic interaction. The shadow area represents the most synergistic dose-window in a dose-response matrix.

**Supplementary tables**

| plasmid name | Genotypes | Origin | Genebank accession | Restriction sites for cloning | Genome size (bp) |
| --- | --- | --- | --- | --- | --- |
| pcDNA3.1-HDV-1 | 1 | Ethiopia | [U81989](https://www.ncbi.nlm.nih.gov/nuccore/U81989) | HindllI and Pst1 | 1679 |
| pcDNA3.1-HDV-2 | 2 | Japan | [X60193](https://www.ncbi.nlm.nih.gov/nuccore/X60193) | Kpnl and EcoRI | 1683 |
| pcDNA3.1-HDV-3 | 3 | Peru | [L22063](https://www.ncbi.nlm.nih.gov/nuccore/L22063) | Hindlll and EcoRI | 1677 |
| pcDNA3.1-HDV-4 | 4 | Japan | [AB118847](https://www.ncbi.nlm.nih.gov/nuccore/AB118847) | Hindlll and EcoRI | 1678 |
| pcDNA3.1-HDV-5 | 5 | Guinea-Bissau | [AM183331](https://www.ncbi.nlm.nih.gov/nuccore/AM183331) | Hindlll and EcoRI | 1687 |
| pcDNA3.1-HDV-6 | 6 | Nigeria | [AM183329](https://www.ncbi.nlm.nih.gov/nuccore/AM183329) | Hindlll and Pst1 | 1680 |
| pcDNA3.1-HDV-7 | 7 | Cameroon | [AM183333](https://www.ncbi.nlm.nih.gov/nuccore/AM183333) | Hindlll and EcoRI | 1674 |
| pcDNA3.1-HDV-8 | 8 | Ivory Coast | [AM183327](https://www.ncbi.nlm.nih.gov/nuccore/AM183327) | Hindlll and Notl | 1681 |

**Table S1 The list of HDV plasmids used in this study**

**Table S2 The list of RT-qPCR primers used in this study**

| **Primer name** | **Sequence 5’-3’** |
| --- | --- |
| DDX58-F | CACCTCAGTTGCTGATGAAGGC |
| DDX58-R | GTCAGAAGGAAGCACTTGCTACC |
| CXCL11-F | AAGGACAACGATGCCTAAATCCC |
| CXCL11-R | CAGATGCCCTTTTCCAGGACTTC |
| CX3CL1-F | ACAGCACCACGGTGTGACGAAA |
| CX3CL1-R | AACAGCCTGTGCTGTCTCGTCT |
| NT5C3A-F | CATCCCAATGTCAAAGTTGTGTCC |
| NT5C3A-R | TCTGTATTCCTCAAGGCACCATC |
| TNFSF10-F | TGGCAACTCCGTCAGCTCGTTA |
| TNFSF10-R | AGCTGCTACTCTCTGAGGACCT |
| SAMHD1-F | CTCGCAACTCTTTACACCGTAGA |
| SAMHD1-R | TTTCCTCCAGCACCTGTAATCTC |
| CD38-F | TCTTGCCCAGACTGGAGAAAGG |
| CD38-R | TGGACCACATCACAGGCAGCTT |
| OR56B1-F | TGTCACTCCAGTGCCTGTGCTT |
| OR56B1-R | CCAGAACCAACTGGCAAATGCTG |
| XRN1-F | CCAGCAAAGCAGTCGTGGAGAA |
| XRN1-R | CCACGACTCTAGCTTCCTCAAG |
| ZC3HAV1-F | GATGATGCTGACCCAAGAGTAGC |
| ZC3HAV1-R | GGACAACCCTTACACAGATGGTC |
| USP41-F | TGAATGTGGACTTCGCCAGG |
| USP41-R | ATGTTGGACAAACAGGGGCA |
| SERPINB1-F | AGCTCAGCATGGTCATCCTGCT |
| SERPINB1-R | CGAGATTCTCAGGTTTAGTCCAC |
| TMEM62-F | GATGCCACTGTAAATCCAGGGC |
| TMEM62-R | ATGGTTGCTCCGACTGCTTTCC |
| CD274-F | TGCCGACTACAAGCGAATTACTG |
| CD274-R | CTGCTTGTCCAGATGACTTCGG |
| MLKL-F | TCACACTTGGCAAGCGCATGGT |
| MLKL-R | GTAGCCTTGAGTTACCAGGAAGT |
| MDA5-F | GCTGAAGTAGGAGTCAAAGCCC |
| MDA5-R | CCACTGTGGTAGCGATAAGCAG |
| IFI44-F | GTGAGGTCTGTTTTCCAAGGGC |
| IFI44-R | CGGCAGGTATTTGCCATCTTTCC |
| TNFα-F | CTCTTCTGCCTGCTGCACTTTG |
| TNFα-R | ATGGGCTACAGGCTTGTCACTC |
| IL1B-F | CCACAGACCTTCCAGGAGAATG |
| IL1B-R | GTGCAGTTCAGTGATCGTACAGG |
| IL6-F | AGACAGCCACTCACCTCTTCAG |
| IL6-R | TTCTGCCAGTGCCTCTTTGCTG |
| IL12A-F | TGCCTTCACCACTCCCAAAACC |
| IL12A-R | CAATCTCTTCAGAAGTGCAAGGG |
| MIP1B-F | GCTTCCTCGCAACTTTGTGGTAG |
| MIP1B-R | GGTCATACACGTACTCCTGGAC |
| MIP1A-F | ACTTTGAGACGAGCAGCCAGTG |
| MIP1A-R | TTTCTGGACCCACTCCTCACTG |
| IL8-F | GAGAGTGATTGAGAGTGGACCAC |
| IL8-R | CACAACCCTCTGCACCCAGTTT |
| MYD88D-F | GAGGCTGAGAAGCCTTTACAGG |
| MYD88D-R | GCAGATGAAGGCATCGAAACGC |
| TNFSF13B-F | ACCACGCGGAGAAGCTGCCAG |
| TNFSF13B-R | CTGCTGTTCTGACTGGAGTTGC |
| WARS-F | GGACATCATCGCCTGTGGCTTT |
| WARS-R | AGTCGCTGTCAGTGAAGCCGAA |
| ISG20-F | ACACGTCCACTGACAGGCTGTT |
| ISG20-R | ATCTTCCACCGAGCTGTGTCCA |
| RNASEL-F | AAGGCTGTTCAAGAACTACACTTG |
| RNASEL-R | TGGATCTCCAGCCCACTTGATG |
| OAS1-F | AGGAAAGGTGCTTCCGAGGTAG |
| OAS1-R | GGACTGAGGAAGACAACCAGGT |
| IFITM1-F | GGCTTCATAGCATTCGCCTACTC |
| IFITM1-R | AGATGTTCAGGCACTTGGCGGT |
| NPPC-F | CGGTCAGAAGAAGGGCGACAAG |
| NPPC-R | GGTGCTCTTGCAGAAGGCGAG |
| ISG15-F | CTCTGAGCATCCTGGTGAGGAA |
| ISG15-R | AAGGTCAGCCAGAACAGGTCGT |
| Viperin (RSAD2)-F | CCAGTGCAACTACAAATGCGGC |
| Viperin (RSAD2)-R | CGGTCTTGAAGAAATGGCTCTCC |
| CCL5-F | CCTGCTGCTTTGCCTACATTGC |
| CCL5-R | ACACACTTGGCGGTTCTTTCGG |
| IFIT1-F | GCCTTGCTGAAGTGTGGAGGAA |
| IFIT1-R | ATCCAGGCGATAGGCAGAGATC |
| IFIT2 -F | GGAGCAGATTCTGAGGCTTTGC |
| IFIT2-R | GGATGAGGCTTCCAGACTCCAA |
| MXA-F | GGCTGTTTACCAGACTCCGACA |
| MXA-R | CACAAAGCCTGGCAGCTCTCTA |
| TAP1-F | GCAGTCAACTCCTGGACCACTA |
| TAP1-R | CAAGGTTCCCACTGCTTACAGC |
| IFI41-F | AGAAGACGCCTAGTACACCACG |
| IFI41-R | GTTCCAGGTTGAGTCGTCTTTCC |
| ADAR1-F | TCCGTCTCCTGTCCAAAGAAGG |
| ADAR1-R | TTCTTGCTGGGAGCACTCACAC |
| Human-IFNα2a-F | TGGGCTGTGATCTGCCTCAAAC |
| Human-IFNα2a-R | CAGCCTTTTGGAACTGGTTGCC |
| Human-IFN-β-F | ATGACCAACAAGTGTCTCCTCC |
| Human-IFN-β-R | GCTCATGGAAAGAGCTGTAGTG |
| Human-IFN-λ1-F | AACTGGGAAGGGCTGCCACATT |
| Human-IFN-λ1-R | GGAAGACAGGAGAGCTGCAACT |
| Human-IFN-γ-F | GAGTGTGGAGACCATCAAGGAAG |
| Human-IFN-γ-R | TGCTTTGCGTTGGACATTCAAGTC |
| Human-β-actin-F | CCTTCCTGGGCATGGAGTCCTG |
| Human-β-actin-R | GGAGCAATGATCTTGATCTTC |
| HBV total RNA-F | TCAGCAATGTCAACGACCGA |
| HBV total RNA-R | TGCGCAGACCAATTTATGCC |
| HBV pregenomic RNA-F | CTCCTCCAGCTTATAGACC |
| HBV pregenomic RNA-R | GTGAGTGGGCCTACAAA |
| Ferns-HDV-f | GCGCCGGCYGGGCAAC |
| Ferns-HDV-r | TTCCTCTTCGGGTCGGCATG |
| Ferns-HDV-probe | 5'FAM-CGCGGTCCGACCTGGGCATCCG-3'TAM |
